# Focusing on conflict in item-specific adaptive control: Insights from a proportion-neutral manipulation

**DOI:** 10.3758/s13421-026-01878-2

**Published:** 2026-04-08

**Authors:** Giacomo Spinelli, Stephen Lupker

**Affiliations:** 1 Department of Psychology, University of Zurich, Binzmuehlestrasse 14, Box 1, 8050 Zurich, Switzerland; 2 Department of Psychology, University of Western Ontario, London, Ontario N6A 5C2 Canada

**Keywords:** Adaptive control, Reactive control, Conflict adaptation, Stroop

## Abstract

Jacoby et al. (*Psychonomic Bulletin & Review*, *10*, 638–644, 2003) reported that, in Stroop tasks, stimuli that more frequently involve targets combined with a congruent distractor (e.g., the word RED in the color red) produce larger Stroop effects than stimuli that more frequently involve targets combined with an incongruent distractor (e.g., RED in green). This pattern suggests that adaptive control can be item-specific in addition to item-nonspecific, and reactive in addition to proactive (although this conclusion has been challenged). This adaptive-control process has often been assumed to be driven by the conflict associated with incongruent stimuli; however, the typical experimental manipulations investigating this issue allow the facilitation associated with congruent stimuli to also play a role. Here, we modified those manipulations in order to focus exclusively on conflict, removing any impact of congruency facilitation, by contrasting targets presented with either neutral (letter strings) or incongruent distractors. Neutral stimuli were presented more frequently than incongruent ones in the Mostly-Neutral (MN) condition and vice versa in the Mostly-Incongruent (MI) condition. Paralleling the original pattern, Stroop interference was larger in the MN condition, suggesting that item-specific conflict frequency can be used to adapt attention accordingly. Importantly, this effect was replicated after experimentally controlling for stimulus frequency, a confound that was found to explain part, but not all, of the general pattern. These results support Jacoby et al.’s claims that (a) control can be adapted in an item-specific fashion and (b) conflict plays a key role in that process.

**Supplementary Information:**

The online version contains supplementary material available at https://doi.org/10.3758/s13421-026-01878-2.

## Introduction

People are vulnerable to influence from irrelevant but salient information. A demonstration of this phenomenon can be found in the classic Stroop (1935) task, in which participants are shown color names printed in colored ink and are required to respond to the less salient component (the target, the ink color) while ignoring the more salient component (the distractor, the color name). “Stroop interference” refers to slower and/or less accurate responses when the distractor is incongruent with the target (e.g., the word BLUE in red color) compared to when the distractor is neutral (e.g., XXXX in red). “Stroop facilitation” refers to faster and/or more accurate responding when the distractor is congruent with the target (e.g., RED in red) compared to when the distractor is neutral. The overall Stroop effect refers to superior performance when word and color are congruent compared to when they are incongruent (MacLeod, 1991).

Is there something people can do to reduce the influence of irrelevant but salient information on performance? One approach to assessing how such adaptive control processes might work has involved use of the Proportion-Congruent (PC) paradigm (Bugg & Crump, 2012). This paradigm was introduced by Logan and Zbrodoff (1979; see also Shor, 1975) together with the list-wide PC procedure. Their results were that the Stroop effect was much larger when subjects were to name colors on a Mostly-Congruent (MC) list than on a Mostly-Incongruent (MI) list. Subsequent research yielded evidence of a similar pattern for Stroop interference (incongruent vs. Neutral) and sometimes but not always for Stroop facilitation (congruent vs. neutral) (e.g., Bugg et al., 2011b; Tzelgov et al., 1992; for a review, see Braem et al., 2019). The general conclusion is that the effects of an irrelevant but salient dimension can be attenuated when most test items are incongruent.

The list-wide PC effect has typically been interpreted as reflecting the action of a general, “item-nonspecific” adaptive-control process (Bugg & Crump, 2012). This process is presumed to increase processing selectivity (i.e., the ability to select task-relevant from task-irrelevant information) as the proportion of incongruent stimuli (stimuli for which the distractor interferes with target processing) in a list increases, such as in an MI list.

Importantly, what makes this adaptive-control process “item-nonspecific” is the idea, implemented in popular cognitive-control models that have been used to simulate the list-wide PC effect (Botvinick et al., 2001), that the process would be applied regardless of the identity of the specific stimuli appearing in either list (e.g., whether the current stimulus has RED as the word component or blue as the color component). The same idea implies that the control state in either list can be set up in advance of stimulus presentation – a *proactive* (i.e., preparatory) form of control. As a result, even though this form of control is only assumed for the MI list (because in the MC list control would actually be in a “reactive” state, with potential interference being handled only when it occurs, with no advance preparation for it), these ideas/effects have led cognitive-control researchers to associate the list-wide PC manipulation with proactive control (Braver, 2012; Gonthier et al., 2016).

### The item-specific Proportion-Congruent (PC) manipulation and its legacy

A pivotal moment in our understanding of adaptive control, however, came with a paper that sent many of these ideas into turmoil – Jacoby et al. (2003). To be more precise, Jacoby et al. (2003) replicated, extended, and popularized a manipulation first developed by Jacoby et al. (1999) (see also Trainham et al., 1997). The manipulation consisted of a simple but clever modification of the PC manipulation, with congruency proportion being manipulated at the level of individual stimuli rather than at the list level. Specifically, half of the words in a Stroop task – the MC items – were mainly presented in their congruent color (e.g., the words RED and BLUE presented in their corresponding colors) whereas the other half – the MI items – were mainly presented in an incongruent color (e.g., the words GREEN and YELLOW presented in their non-corresponding colors). What makes this manipulation different from a list-wide one is that both MC and MI items were intermixed in the same list – a 50:50 congruent/incongruent list. However, similar to the list-wide PC effect, an item-specific PC effect emerged, with a larger congruency effect (resulting from both larger facilitation and larger interference) for MC items than for MI items.

This pattern, primarily emerging in response latencies in a regular Stroop task, was subsequently extended by Jacoby’s group. First, they examined the pattern using a process-dissociation procedure (Jacoby, 1991; Lindsay & Jacoby, 1994), which allowed them to conclude that the item-specific PC effect mainly resulted from a greater contribution of word reading to performance on MC than MI items (Jacoby et al., 1999, 2003; see also Trainham et al., 1997). Second, using a block analysis, Jacoby et al. (2003) showed that the item-specific PC effect emerges early on in the experiment and does not seem to change as a function of time on task.

More importantly, the mere *existence* of an item-specific PC effect was sufficient to change how adaptive control was thought about and studied. As noted, the only PC manipulation used prior to Jacoby et al. (2003) was the list-wide one, the impact of which was mainly thought to reflect the action of an item-nonspecific process (Bugg & Crump, 2012). The fact that an item-specific PC manipulation produced a similar impact suggested not only that item-specific processes could also be used, but that the PC effect produced by list-wide PC manipulations might have reflected such processes all along. The reason is that, in standard list-wide PC manipulations, list-wide and item-specific congruency proportion are confounded with one another. Specifically, the items in an MC list are all “MC items” (e.g., all words in that list appear most often in their congruent color) and the items in an MI list are all “MI items” (e.g., all words in that list appear most often in incongruent colors). As a result, to maintain the conclusion that the list-wide PC effect reflects item-nonspecific control, Jacoby et al.’s (2003) results required a revisitation of list-wide manipulations in order to dissociate the impact of the list-wide versus item-specific congruency proportions.

Jacoby (see Bugg et al., 2008) contributed to making this dissociation possible with a design which would later become the most popular and recommended procedure (see Braem et al., 2019; for alternative dissociation procedures, see Spinelli et al., 2019; Viviani et al., 2024). In their design, the stimuli used are divided into two separate sets: the inducer items, for which congruency proportion is manipulated directly (as is typically done for all stimuli in standard list-wide PC paradigms), and the diagnostic items, which have a fixed 50:50 congruent/incongruent ratio and are identical in the two lists. The critical aspect of this manipulation is that, while list-wide and item-specific congruency proportion are confounded with one another for inducer items (as is typically the case for all stimuli in standard list-wide PC paradigms), such is not the case for diagnostic items, for which only list-wide congruency proportion (and hence, item-nonspecific control) might have an impact.

Examination of performance on the diagnostic items has provided a more complex picture of item-nonspecific control than originally thought. On the one hand, those items do tend to produce a PC effect (Spinelli & Lupker, 2023, 2024; but not always, see Blais & Bunge, 2010; Bugg, 2014; Bugg et al., 2008). On the other hand, that effect is consistently much smaller than the one produced by inducer items in that manipulation or all stimuli in a standard list-wide PC manipulation. The implication is that item-nonspecific control, as documented by the performance with the diagnostic items, likely plays a less important (although not null) role in adaptive control than originally assumed in models based entirely on item-nonspecific control (e.g., Botvinick et al., 2001; for an alternative based entirely on item-specific control, see Blais et al., 2007).

### Processes involved in the item-specific PC effect

Perhaps the most productive contribution of Jacoby et al. (2003) comes from the research carried out to resolve the competition between the two explanations they proposed for the item-specific PC effect (see also Schmidt & Besner, 2008). The first explanation is that the effect might be an item-specific version of the adaptive-control process described above for the list-wide PC effect. That is, the effect could reflect a state of reduced selectivity for MC items, leading to greater interference from incongruent distractors when they appear (and possibly, greater facilitation from the frequent congruent distractors) as well as a state of increased selectivity for MI items, leading to reduced interference from incongruent distractors (and, possibly, reduced facilitation from congruent distractors). Different from the item-nonspecific process, however, this process would not involve any proactive preparation for upcoming stimuli, because any selectivity adjustment would have to be made once the item has been identified, in a *reactive* fashion (Gonthier et al., 2016).

The second explanation proposed by Jacoby et al. (2003) for the item-specific PC effect is that it reflects a contingency-learning process whereby participants learn to associate specific words with the response typically made to stimuli containing those words. Because in the standard item-specific PC manipulation the high-contingency (i.e., typical) response is the congruent one for an MC word (e.g., the response red for the word RED) versus an incongruent one for an MI word (e.g., the response yellow for the word GREEN), it follows that responses will be faster in those situations than in situations requiring low-contingency (i.e., atypical) responses (i.e., for MC words requiring an incongruent response and for MI words requiring the congruent response). The result would be a larger congruency effect for MC items (for which contingency learning will increase the difference between congruent and incongruent stimuli) than for MI items (for which contingency learning will reduce the difference between congruent and incongruent stimuli). Importantly, because invoking a process of this sort would not depend on the words being color names (i.e., the words would not have to be strongly distracting stimulus components), it would be distinct from processes of the adaptive-control kind. This contingency-learning explanation was subsequently developed into a larger alternative account of item-specific PC effects (Schmidt & Besner, 2008; Schmidt et al., 2010) and PC effects more generally (Schmidt, 2013a, 2019).

Jacoby et al. (2003) favored neither of these explanations, because in their design, item-specific adaptive control and contingency learning are perfectly confounded. Further, it was later noted that, in that design, a potential item-specific adaptive-control process could actually be due to either of two processes (Bugg et al., 2011a). First, it could be due to a *distractor*-specific adaptive-control process based on the distinction between MC and MI distractors (i.e., the words themselves). Second, it could be due to a *target*-specific adaptive-control process based on the distinction between MC and MI targets (i.e., the colors). Jacoby et al.’s (2003) manipulation did not allow a dissociation between those processes because, in that manipulation, MC items were stimuli which combined an MC color (e.g., the color red presented with RED more often than with BLUE) and an MC word (e.g., the word RED presented in red more often than in blue). Similarly, MI items were stimuli which combined an MI color (e.g., the color yellow presented with GREEN more often than with YELLOW) and an MI word (e.g., the word GREEN presented in yellow more often than in green).

This distinction between target- and distractor-specific adaptive control is particularly important because, as Bugg et al. (2011a) recognized, whereas a contingency-learning account could compete with an account based on distractor-specific adaptive control as a potential explanation of the item-specific PC effect, it could not compete with an account based on target-specific adaptive control. The reason is that the (only) contingencies that vary between MC and MI items are *distractor*-response ones (e.g., between the *word* RED and the response red)*. Target*-response contingencies (e.g., between the *color* red and the response red) do not vary. That is, each target in a Stroop task, either of the MC or the MI type, always has a 1:1 association with its required response (e.g., the color red will always require the response red). As a result, a target-specific adaptive-control process would not be explainable within a contingency-learning account.

Based on these considerations (and others which are not relevant for present purposes), Bugg et al. (2011a) introduced a number of modifications in the item-specific PC manipulation meant to dissociate adaptive-control from contingency-learning processes. For present purposes, the most important modification was the introduction of a distinction between inducer stimuli, for which the congruency proportion was manipulated, and diagnostic stimuli, which have a fixed 50:50 congruent/incongruent ratio and play the crucial role for determining the nature of the processes involved.

Although Bugg et al. (2011a) introduced this distinction in the context of a picture-word Stroop task, a similar distinction was later applied to color-word Stroop tasks as well. For example, Bugg and Hutchison (2013), in one version of their experiments, presented MC and MI colors (with the corresponding words – the inducer stimuli) in the first part of the experiment. In the second part, the same colors were presented with a new set of (incongruent) words – the diagnostic stimuli – which create a control for contingency learning because no contingencies had been learned for those words in the first part of the experiment. Crucially, latencies for those incongruent words were slower when presented with MC than MI colors, consistent with the idea that participants had learned to adapt selectivity based on the colors’ identity in the first part of the experiment (but for a counterargument, see Schmidt, 2013a, 2019).

Research in our lab has later replicated this pattern of selective adaptation in experiments that mixed inducer and diagnostic stimuli in the same block, and in which the MC/MI distinction was based not only on the identity of the targets (e.g., colors) but also on that of distractors (e.g., words), while controlling for contingency learning (Spinelli & Lupker, 2020, 2024; Spinelli et al., 2022; but see Atalay & Misirlisoy, 2012). The more general point, in any case, is that several studies now suggest that item-specific adaptive control, like its item-nonspecific counterpart, might be a more robust phenomenon than originally thought (Braem et al., 2019; see also Gonthier et al., 2016, Tang et al., 2023).

### The present research

In sum, since the item-specific PC paradigm was introduced, many issues concerning the underlying processes have been addressed, particularly the processes of the adaptive-control kind. (Note that these issues have many ramifications that we have not discussed, e.g., how item-specific adaptive control interacts with the item-nonspecific type: Bozkurt et al., 2023; Hutchison, 2011; whether this type of control is dependent on limited-capacity resources, as the idea that item-specific control reflects a reactive process would imply: Ileri-Tayar et al., 2025; Spinelli et al., 2020; Suh & Bugg, 2021; etc.)

An issue that has been less pursued, however, is the role of *conflict* in the item-specific PC effect. That is, there seems to be no straightforward demonstration in the literature that the effect results, at least in part, from the infrequently versus frequently *conflicting* nature of MC and MI items, respectively. The reason is that, in all relevant studies, not only the proportion of stimuli involving conflict (i.e., incongruent) but also that of stimuli involving facilitation (i.e., congruent) have been manipulated. The implication is that, even when appropriate controls are in place for processes unrelated to adaptive control, the pattern assumed to reflect item-specific adaptive-control (if one is obtained) would not necessarily be conflict driven. Indeed, the pattern might be mainly (or entirely) facilitation driven, with frequent facilitation for MC items leading to reduce selectivity in order to better exploit such facilitation and infrequent facilitation for MI items having the opposite effect because the opportunities for facilitation that those items offer are more limited.

Conflict, however, has typically been thought of as the main trigger of adaptive-control processes, in PC manipulations as well as in others (e.g., Gratton et al., 1992). For example, in Botvinick et al.’s (2001) *conflict*-monitoring model, it is detection of conflict between the target and the distractor which drives control adaptation. Similar considerations naturally apply to models derived from the conflict-monitoring model, such as Blais et al.’s (2007) item-specific variation of the model. Indeed, the fact that adaptive-control processes are often called “conflict-adaptation” effects (e.g., Schmidt et al., 2015) is a telltale sign of the role conflict is assumed to play in such effects. (Here, consistent with Braem et al., 2019, we will use the phrase “adaptive control” to refer to a type of control that could be either conflict driven, facilitation driven, or both, and the phrase “conflict adaptation” to refer to a type of adaptive control that could only be conflict driven.)

Although the role of facilitation has often been neglected, there is evidence that facilitation influences “conflict-adaptation” effects. For example, Bugg et al. (2011b) conducted a list-wide manipulation involving not only congruent and incongruent but also *neutral* stimuli such that a Mostly-Neutral (MN) list was created in addition to regular MC and MI ones. This addition makes it possible to use the proportion manipulation to investigate the impact of facilitation-driven control (by contrasting the MC and the MN list) from that of interference-driven control (by contrasting the MI and the MN list). Interestingly, although the MI/MN contrast did reveal an impact of conflict-driven control, the MC/MN contrast revealed an equally large (if not larger) impact of facilitation-driven control (for similar evidence from another adaptive-control paradigm, see Compton et al., 2012).

Building on this approach, we developed a simpler but more straightforward manipulation (Spinelli & Lupker, 2021). In this *list-wide* proportion-*neutral* (PN) manipulation, implemented in the color-word Stroop task, we eliminated congruent stimuli entirely and contrasted interference effects (incongruent – neutral) for an MN list versus an MI list. Doing so not only has the advantage of focusing on conflict – the active ingredient of conflict adaptation – by using a conflict frequency manipulation (rather than a conflict-and-facilitation frequency manipulation, as with PC manipulations), but also effectively controls for contingency learning because, as long as each distractor used is combined with at least two targets with equivalent frequency, no (distractor-response) contingencies can be learned. In addition, improving on previous list-wide PN manipulations (Goldfarb & Henik, 2007; Tzelgov et al., 1992), we also controlled for the potential impact of item-specific conflict adaptation. This control was made possible by dividing the stimuli into an inducer set for which both item-specific and item-nonspecific conflict adaptation could produce a list-wide PN effect (i.e., a larger interference effect in the MN list than in the MI list) and a diagnostic set for which only item-nonspecific adaptive control could produce such an effect.

Spinelli and Lupker’s (2021) central result was that a PN effect did indeed emerge for diagnostic items, providing evidence of what could legitimately be called an item-nonspecific conflict-adaptation effect. Interestingly, however, inducer items also produced a PN effect that was much larger (55 ms) than the one the diagnostic items produced (24 ms). Such a discrepancy would seem to be attributable to item-specific conflict adaptation, the only additional process that inducer items allowed and diagnostic items did not (i.e., contingency learning would not have been possible in this experiment).

The present experiments were aimed at providing a more direct demonstration of the usefulness of a PN manipulation in the study of item-specific conflict adaptation. To that end, we designed an item-specific version of our PN manipulation contrasting MN and MI colors presented with either incongruent color names or neutral letter strings (Experiment 1). Although that version effectively controlled for all of the confounds discussed up to this point in the literature for the item-specific PC effect (and did so without requiring a distinction between inducer and diagnostic items), we determined that it likely contained a new confound. Focusing on this confound in a separate experiment (Experiment 2) revealed the necessity to control for it in order to obtain clear evidence of item-specific conflict adaptation. We designed such a control and obtained evidence for item-specific conflict-adaptation in a final experiment (Experiment 3) using an inducer/diagnostic design. In sum, this research provides the first piece of evidence for such conflict adaptation, a process that has often been assumed, but has never yet been fully demonstrated, in research inspired by Jacoby et al.’s (2003) seminal work.

## Experiment 1

This experiment involved a PN manipulation similar to that conducted for inducer items in Spinelli and Lupker (2021), except that MN and MI items, defined by their target identities, were intermixed in the same list (i.e., an item-specific PN manipulation; note that it would be more precise to call the manipulation “target-specific” but, to avoid confusion, we will call the manipulation and the effect associated with it “item-specific” in the following unless it is necessary to specify the nature of the manipulation/effect). We also reduced the stimulus set to the minimum necessary for such a manipulation, i.e., from six to four colors and from 12 written stimuli (either color names or letter strings) to eight. Our hypothesis was that an item-specific PN effect, similar to the list-wide PN effect reported by Spinelli and Lupker, would emerge, with a larger interference effect for MN than MI targets.

### Method

#### Participants

The sample size for this experiment was determined based on the list-wide PN neutral effect produced by inducer items in Spinelli and Lupker’s (2021) experiment, ${\eta }_{p}^{2}$ =.263. The reason is that the inducer items in that experiment, as discussed, are the ones that potentially involved item-specific conflict adaptation. An a priori power analysis was performed with G*Power 3.1 (Faul et al., 2009) treating the PN effect as an ANOVA within-subject factor and specifying ${\eta }_{p}^{2}$ =.263 as the effect size with the “as in SPSS” option. The analysis revealed that 25 participants would be necessary for a power of.80 to detect an effect of that size. We decided to collect at least 30. Thirty-two students at the University of Western Ontario (age 18–21 years) participated for course credit. All participants reported being native English speakers and having normal or corrected-to-normal vision.

#### Materials

Four color names (RED, BLUE, GREEN, YELLOW) and four letter strings (FFF, KKKK, JJJJJ, HHHHHH) were used as distractors and the colors corresponding to the color names (red [R: 255; G: 0; B: 0], blue [R: 0; G: 0; B: 255], green [R: 0; G: 192; B: 0], yellow [R: 255; G: 255; B: 0]) were used as target colors. The four targets were divided into two sets: One included the colors red and blue, which were only combined with GREEN and YELLOW to create incongruent stimuli, and their length-matched letter strings (JJJJJ, HHHHHH) to form neutral stimuli; the other included the colors green and yellow, which, similarly, were only combined with RED and BLUE to create incongruent stimuli, and their length-matched letter strings (FFF, KKKK) to create neutral stimuli.

Consistent with Spinelli and Lupker (2021), we used letter strings for neutral stimuli, a widely used baseline that is assumed to allow the separation of facilitation from interference in the Stroop effect (MacLeod, 1991). It must be noted that the contrast between incongruent color names and letter strings likely involves conflict at more than one level, for example, between the color-naming task and the word-reading task (with the latter being possible for incongruent color names but not for letter strings), and between the information the target and the distractor convey (which is directly incompatible for incongruent color names but not for letter strings; Parris et al., 2022). However, since we were not interested in the nature of the conflict involved in the incongruent–neutral contrast, these distinctions were irrelevant for present purposes.

Two versions of the experiment, counterbalanced across participants, were created. In one version, represented in Table 1, one of the two target sets (e.g., the one including the colors red and blue) served as the MN set and the other (e.g., the one including the colors green and yellow) served as the MI set. In the other version, these assignments were swapped. In the MN set, each color was presented 12 times with each of the two incongruent color names and 36 times with each of the two letter strings associated with that color. The reverse was true in the MI set, with each color being presented 36 times with each of the two incongruent color names and 12 times with each of the two letter strings associated with that color. As a result, the target-specific proportion of incongruent stimuli was 25% for MN colors and 75% for MI colors. The list-wide proportion of incongruent stimuli, however, was 50%, as there were 192 incongruent and 192 neutral stimuli overall (384 stimuli in total).

**Table 1:** Template for the frequency of target-distractor combinations in Experiment 1

		Distractor							
Set	Target	RED	GREEN	FFF	JJJJJ	BLUE	YELLOW	KKKK	HHHHHH
MN	Red		12		36		12		36
	Blue		12		36		12		36
MI	Green	36		12		36		12	
	yellow	36		12		36		12	

*MN* Mostly Neutral, *MI* Mostly Incongruent

Further, the *distractor*-specific proportion of incongruent stimuli was entirely determined by the color-name versus letter-string nature of the written stimulus (i.e., all color names were 100% incongruent and all letter strings were 0% incongruent). This fact might have produced a bias for participants in our experiment to adapt control based on the distractor stimulus component. Potentially, the result of this process would be a reduction of interference effects because, while being able to determine that a stimulus is neutral should have little or no effect on that stimulus, being able to determine that a stimulus is incongruent should lead to heightened selectivity and hence a speed up for responses to that stimulus. Note, however, that this fact about the present experiment was true in Spinelli and Lupker’s (2021) experiment as well (i.e., all color names were also 100% incongruent and all letter strings were also 0% incongruent in that experiment); however, the interference effect in that experiment was quite large (over 100 ms overall; see also Stroop, 1935). Thus, the fact that distractor-specific adaptive control is possible does not seem to have a strong impact in this paradigm (and should not prevent target-specific adaptive control in any case; Spinelli et al., 2022).

Finally, as mentioned, contingency learning could not be used in this design because each distractor appeared with each of the two targets associated with it an equal number of times (either 12 or 36), meaning that there was no high- or low-contingency response associated with any of the distractors used. For this reason, in this manipulation unlike in more typical item-specific PC manipulations (e.g., Bugg & Hutchison, 2013), there was no need to distinguish between inducer and diagnostic items in order to control for contingency learning.

#### Procedure

Each trial began with a fixation symbol (“+”) displayed for 250 ms in the center of the screen followed by a colored word/letter string displayed for 2,000 ms or until the participant’s response, which was recorded with a microphone connected to the testing computer. Participants were instructed to name the color as quickly and as accurately as possible while ignoring the word/letter string itself. Stimuli were presented in uppercase Courier New font, pt. 14, against a medium gray background (R: 128; G: 128; B: 128). No feedback was provided. The experiment was divided into two equal-sized blocks (192 trials per block) with a self-paced pause in the middle. The order of trials within each block was randomized. Initially, participants performed a practice session including eight trials in which a string of Xs (“XXXX”) was presented twice in each of the four colors used in the experiment. The experiment was run using DMDX (Forster & Forster, 2003) software. This experiment and the following were performed in line with the principles of the Declaration of Helsinki. Approval was granted by the Research Ethics Board of the University of Western Ontario (protocol # 108956).

## Results

**Fig. 1:**
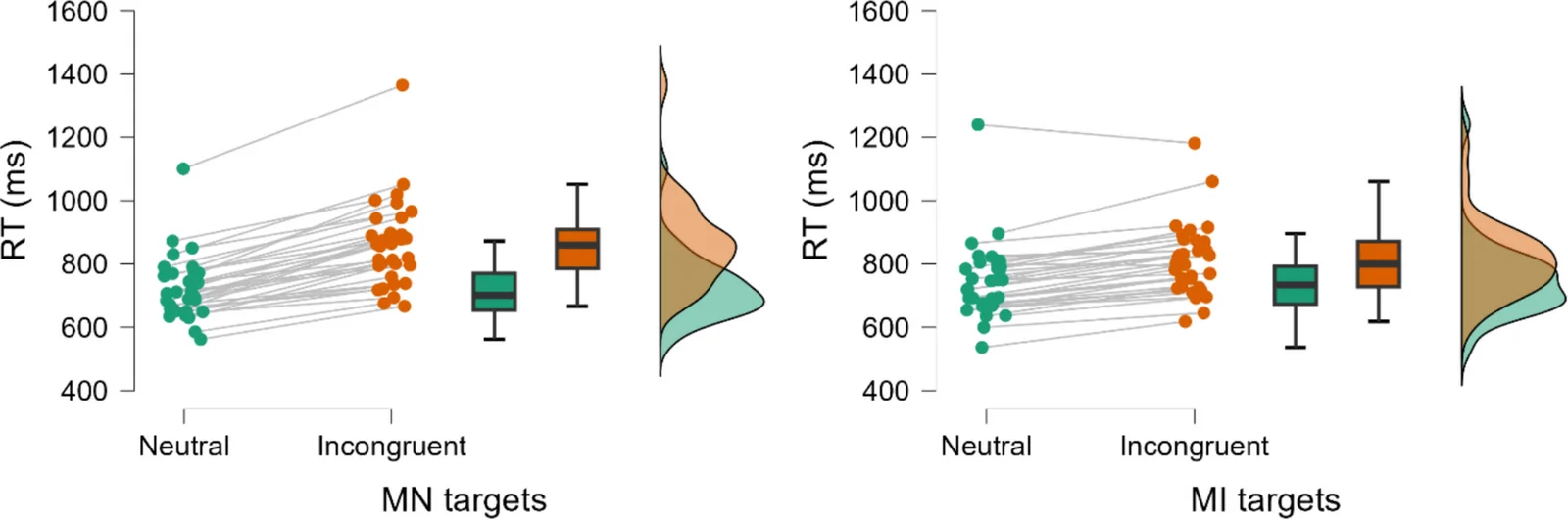
Raincloud plots of participants’ mean response times (RTs) in Experiment 1. MN = Mostly Neutral; MI = Mostly Incongruent. See the online version of this article for colors

The waveforms of responses were manually inspected with CheckVocal (Protopapas, 2007) to determine the accuracy of the response and the correct placement of timing marks. Prior to the analyses, invalid trials due to technical failures and responses faster than 300 ms or slower than the time limit (accounting for 1.1% of the data) were discarded. No participant was excluded because all produced a correct response on at least 75% of the trials. These criteria were specified in advance and are consistent with previous work in our lab (e.g., Spinelli & Lupker, 2020, 2021, 2023, 2025). Analyses were performed with JASP version 0.19.3 (JASP Team, 2025). Participants’ mean response times (RTs) for correct responses and error rates were analyzed using an ANOVA with Congruency (Neutral vs. Incongruent) and Target type (Mostly neutral vs. Mostly incongruent) as within-subject factors. The mean RTs and error rates are presented in Table 2, and raincloud plots of participants’ mean RTs (the typically most sensitive dependent measure in PC paradigms) are presented in Fig. 1.

**Table 2:** Mean response times (RTs) and percentage error rates (and corresponding 95% confidence intervals for effect sizes) in Experiment 1

	Target type					
	RTs			Error rates		
Congruency	MN	MI	Target type effect	MN	MI	Target type effect
Neutral	722 [686, 758]	744 [701, 788]	22 [7, 38]	0.6 [0.2, 1.1]	0.5 [0.0, 0.9]	−0.1 [−0.7, 0.4]
Incongruent	860 [811, 910]	811 [771, 852]	−49 [−28, −70]	3.3 [1.3, 5.2]	2.5 [1.6, 3.4]	−0.8 [−2.3, 0.8]
Interference effect	138 [118, 159]	67 [52, 83]	−71 [−48, −94]	2.7 [1.0, 4.3]	2.0 [1.1, 3.0]	−0.7 [−2.1, 0.9]

*MN* Mostly Neutral, *MI* Mostly Incongruent

*Response times (RTs)*. There was a main effect of Congruency, *F*(1, 31) = 219.45, *MSE* = 1537.35, *p* <.001, ${\eta }_{p}^{2}$ =.876, indicating an interference effect, with faster responses to neutral than incongruent stimuli. The main effect of Target Type was marginal, *F*(1, 31) = 3.36, *MSE* = 1659.06, *p* =.076, ${\eta }_{p}^{2}$ =.098, indicating that responses were slower to MN than MI targets overall, but the difference was not statistically significant. More importantly, there was a significant interaction between Congruency and Target type, *F*(1, 31) = 39.42, *MSE* = 1028.27, *p* <.001, ${\eta }_{p}^{2}$ =.560, reflecting the typical pattern of item-specific PC effects, with a larger interference effect for MN targets (138 ms, 95% confidence interval (CI) [118, 159]) than for MI targets (67 ms, 95% CI [52, 83]). A simple main effect analysis revealed that this interaction resulted from both a speed-up for neutral stimuli appearing with MN versus MI targets, *t*(31) = −2.91, *p* =.007, ${\eta }_{p}^{2}$ =.214, and a slow-down for incongruent stimuli appearing with MN versus MI targets, *t*(31) = 4.68, *p* <.001, ${\eta }_{p}^{2}$ =.414.

*Error rates*. There was a main effect of Congruency, *F*(1, 31) = 19.21, *MSE* =.001, *p* <.001, ${\eta }_{p}^{2}$ =.383, indicating an interference effect, with more accurate responses to neutral than incongruent stimuli. Neither of the other effects was significant, both *p*s ≥.293.

## Discussion

The results of Experiment 1 were clear-cut: MN targets produced a larger interference effect than MI targets in the latencies – an item-specific *PN* effect similar to the more typical item-specific *PC* effect emerging in item-specific PC manipulations (Jacoby et al., 2003). Unlike the item-specific PC effect, however, this effect seems to allow the conclusion that the underlying process must be a conflict-adaptation one because the only difference between MN and MI targets was in the frequency with which conflict (as opposed to conflict *and* facilitation) occurred for those target types.

A closer look at the item-specific PN effect, however, reveals a potential problem for this conclusion: The effect was driven not only by incongruent stimuli but also by neutral ones. Specifically, not only were responses to incongruent stimuli slower when those stimuli were presented with MN versus MI targets, but responses to *neutral* stimuli were faster when those stimuli were presented with MN versus MI targets. The pattern for incongruent stimuli is consistent with a conflict-adaptation process because responses to stimuli involving conflict should be slower when selectivity is lower (with MN targets) than when it is higher (with MI targets). The pattern for neutral stimuli, however, is harder to reconcile with a conflict-adaptation process because the state of selectivity should be inconsequential for stimuli not involving conflict or facilitation.

Note that such is not necessarily the case in situations other than that created by the present manipulation. For example, in item-specific *PC* manipulations, it is usual for *congruent* stimuli to be faster with MC versus MI items (e.g., Jacoby et al., 2003; but see, e.g., Bugg et al., 2011a). However, because those stimuli potentially involve facilitation, that pattern could be attributed to participants taking more advantage of that facilitation when selectivity is lower (with MC items) than when it is higher (with MI items).

Note, however, that there is a potential explanation for why *neutral* stimuli like the ones we used could be affected by proportion manipulations, although, typically, this explanation applies to manipulations of the list-wide kind (but for an exception, see Schmidt et al., 2014). Indeed, in Spinelli and Lupker’s (2021) list-wide manipulation, similar to the present experiment, we did find faster responses to neutral stimuli in the MN versus MI list (in addition to slower responses to incongruent stimuli in the MN vs. MI list) for both inducer and diagnostic items. Although, as noted, that pattern is inconsistent with a conflict-based process, it is consistent with a *time*-based process. Specifically, it is consistent with the idea that participants in speeded tasks establish a time criterion for response emission (i.e., the point in time at which they expect, and will attempt, to respond) depending on the characteristics of the stimuli in the list (the *time-criterion account*, Lupker et al., 1997; for a discussion of why this account should be favored over Schmidt’s (2013b) temporal-learning account, a similar time-based account, see Spinelli & Lupker, 2022; Spinelli et al., 2019). When most of the stimuli in a list are relatively easy (e.g., neutral stimuli), the criterion will be set at an earlier point in time. When most of the stimuli in a list are relatively hard (e.g., incongruent ones), the criterion will be set at a later point in time. As a result, responses to the same stimulus, for example, a neutral stimulus, would be faster in a list of the former type (e.g., an MN list) than in a list of the latter type (e.g., an MI list; Lupker et al., 1997; Spinelli et al., 2019; Spinelli & Sulpizio, 2024).

In sum, the pattern of the list-wide PN effect in Spinelli and Lupker (2021) is explainable by invoking, on one hand, a general time-based process which would produce a tendency for slower responses in the MI list and whose impact is apparent with the neutral stimuli. On the other hand, a conflict-based process would also seem to be necessary in order to explain why that tendency is not observed with incongruent stimuli (i.e., why those stimuli are *faster*, rather than slower, in the MI list compared to the MN list). The point, in any case, is that, in the context of a *list-wide* manipulation, a pattern like the one we obtained, one based on the use of neutral stimuli, would be explainable.

With the present *item-specific* manipulation, however, such an explanation would face a problem. The time-criterion account only applies to list-wide manipulations or, at most, sequential ones (Spinelli & Lupker, 2022; Taylor & Lupker, 2001). Because the stimuli being contrasted are intermixed in the same list in an *item-specific* manipulation, such an account would be unable to explain any differences produced in such a manipulation. Specifically, because neutral distractor stimuli presented with MN targets and neutral distractor stimuli presented with MI targets were intermixed, the criterion for response emission would have been essentially equivalent for the two target types. As a result, no difference between those stimuli would be expected from a time-criterion account (the same, of course, holds for incongruent stimuli). Clearly, then, another explanation is needed. In Experiment 2, we tested such an alternative explanation.

## Experiment 2

In designing the item-specific PN manipulation implemented for Experiment 1, we followed current recommendations to control for the typical confound involved in item-specific manipulations – contingency learning (Braem et al., 2019; Schmidt & Besner, 2008). However, in doing so, we introduced another confound, one that has attracted considerably less attention, at least in the context of item-specific manipulations (but see Bugg & Crump, 2012; Crump & Milliken, 2009; Hutcheon & Spieler, 2017). This confound has to do with the frequency with which individual stimuli (i.e., specific target-distractor combinations, e.g., RED in green) appeared across the cells being contrasted. Specifically, among MN targets, neutral stimuli were *individually* three times more frequent than incongruent stimuli (36 vs. 12 occurrences per stimulus), and similarly, for MI targets, neutral stimuli were *individually* three times less frequent than incongruent stimuli (12 vs. 36 occurrences per stimulus). The reason this fact is relevant is that more frequent stimuli might be processed more easily than less frequent ones. That is, the mechanism producing the pattern for neutral stimuli might be stimulus repetition (either of the immediate kind – from one trial to the next – or from more trials back: Cochrane & Pratt, 2022; Gallego et al., 2025), which produces robust effects for any type of stimulus and naturally occurs more frequently for high- than low-frequency stimuli (e.g., Bentin & McCarthy, 1994; see also Logan, 1988). In fact, a stimulus-frequency confound would be able to explain not only the pattern found in Experiment 1 for incongruent stimuli (slower responses in the low-frequency MN condition than in the high-frequency MI condition) but, importantly, also that found for neutral stimuli (faster responses in the high-frequency MN condition than in the low-frequency MI condition) – a pattern that, as discussed, is inconsistent with either conflict-based or time-based processes. In other words, the item-specific PN pattern of Experiment 1 might have been driven, in whole or in part, by the fact that our conflict-frequency manipulation was also a stimulus-frequency one (for a similar argument, see Mayr et al., 2003).^1^

In order to determine the plausibility of this idea, we conducted a number of additional analyses for Experiment 1, reported in the Online Supplementary Materials, using two proxies for stimulus-frequency effects: immediate stimulus repetitions (i.e., repetitions of the target and/or the distractor from the previous trial; Cochrane & Pratt, 2022) and episodic stimulus repetitions (i.e., repetitions of the target from the most recent episode containing the same distractor as that contained in the current trial; Gallego et al., 2025). The results were generally consistent with the idea that stimulus frequency might have partially contributed to the item-specific PN effect found in Experiment 1.

However, recognizing that stimulus frequency can have an impact beyond that of the proxies we used (e.g., Schmidt et al., 2020), in Experiment 2, we aimed to get a fuller picture of the impact stimulus frequency might have had in Experiment 1 by manipulating stimulus frequency independently from item-specific conflict frequency. To do so, we simply changed the stimulus frequencies used in Experiment 1 so that half of the distractors we used (including both the letter strings and the color names) appeared with low frequency and the other half appeared with high frequency. In contrast, each of the targets we used had a 50/50 probability of appearing in a neutral/incongruent stimulus. In other words, in Experiment 2, unlike in Experiment 1, the item-specific congruency proportion was not manipulated, preventing us from examining an item-specific PN effect. On the other hand, this design allowed us to dissociate the impact of stimulus frequency from that of item-specific conflict adaptation and, thus, have an independent measure of stimulus frequency (for a similar approach in the context of the contingency-learning confound in item-specific PC manipulations, see Schmidt & Besner, 2008). Observing a sizeable effect of stimulus frequency in this experiment would have important implications for the interpretation of Experiment 1, as well as that of any study involving a stimulus-frequency confound (potentially, a large number of them, considering, e.g., that many studies present neutral stimuli with a lower frequency than congruent and incongruent ones; see Spinelli & Lupker, 2025).

### Method

#### Participants

Reasoning that the item-specific PN effect in Experiment 1 might potentially have resulted entirely from a stimulus-frequency process, we used the same sample size as in Experiment 1 to detect an impact of that process in the present experiment. Thirty-two (31 after exclusions, see below) students at the University of Western Ontario (age 18–20 years) participated for course credit. All participants reported being native English speakers and having normal or corrected-to-normal vision. None had participated in Experiment 1.

#### Materials

We used the same four color names and four letter strings used in Experiment 1 as distractors and the same four colors as targets. The four targets were divided in the same two sets as in Experiment 1 (red and blue, which were only combined with GREEN, YELLOW, JJJJJ, and HHHHHH, and green and yellow, which were only combined with RED, BLUE, FFF, and KKKK). However, the eight distractors were also divided into two sets: one including RED, GREEN, and their length-matched letter strings (FFF, JJJJJ), the other including BLUE, YELLOW, and their length-matched letter strings (KKKK, HHHHHH).

Two versions of the experiment, counterbalanced across participants, were created. In one version, represented in Table 3, one of the two distractor sets (e.g., the one including RED, GREEN, FFF, and JJJJJ) served as the low-frequency set and the other (e.g., the one including BLUE, YELLOW, KKKK, and HHHHHH) served as the high-frequency set. In the other version, these assignments were swapped. In the low-frequency set, each distractor was presented 12 times with each of the two colors associated with that distractor. In the high-frequency set, each distractor was presented 36 times with each of the two colors associated with that distractor. As a result, low-frequency stimuli were three times less frequent than high-frequency ones.^2^ However, the target-specific proportion of incongruent stimuli was 50% for all the colors. The list-wide proportion of incongruent stimuli was also 50%, with 192 incongruent and 192 neutral stimuli overall (384 stimuli in total). Finally, the distractor-specific proportion of incongruent stimuli, as in Experiment 1, was entirely determined by the color-name versus letter-string nature of the written stimulus (i.e., all color names were 100% incongruent and all letter strings were 0% incongruent). However, as noted for Experiment 1, interference effects are still large under these conditions and, most importantly, this fact does not represent a confound for the frequency manipulation because it applies to both low- and high-frequency distractors.

**Table 3:** Template for the frequency of target-distractor combinations in Experiment 2

Target	Distractor							
	Low frequency				High frequency			
	RED	GREEN	FFF	JJJJJ	BLUE	YELLOW	KKKK	HHHHHH
Red		12		12		36		36
Blue		12		12		36		36
Green	12		12		36		36	
Yellow	12		12		36		36	

#### Procedure

The procedure was the same as in Experiment 1 except for the stimulus set and the fact that the experiment was administered to 31 (instead of 32) valid participants.

## Results

**Fig. 2:**
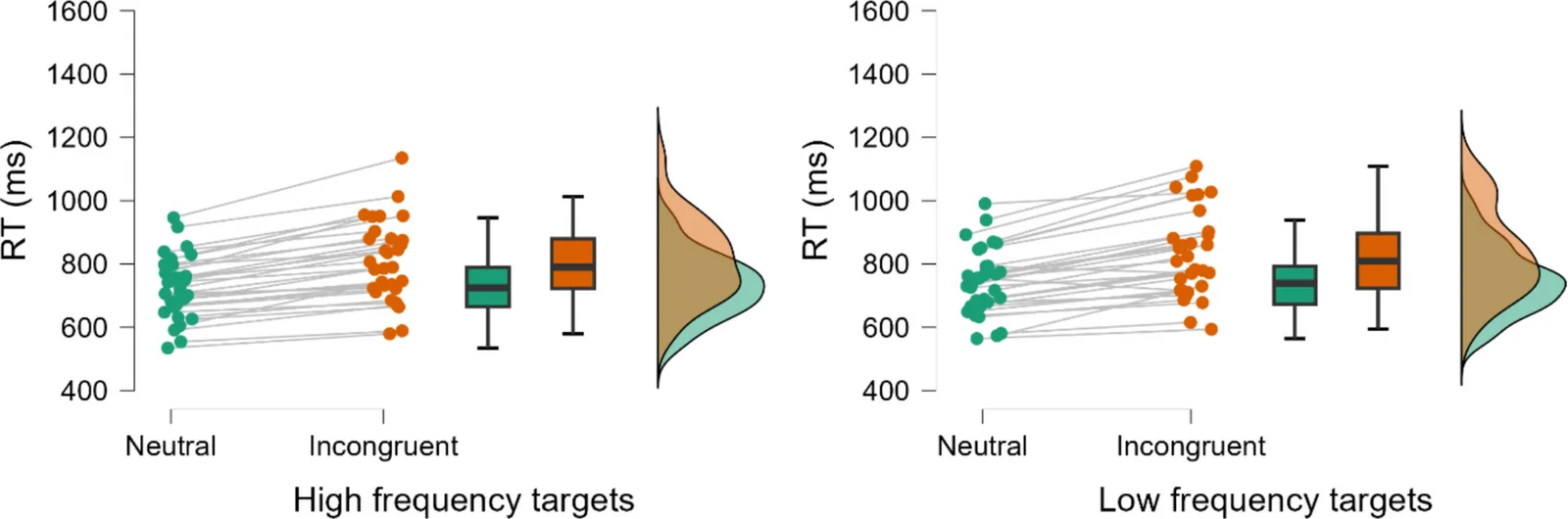
Raincloud plots of participants’ mean response times (RTs) in Experiment 2. See the online version of this article for colors

The data were pre-processed in the same way as for Experiment 1. Prior to the analyses, invalid trials due to technical failures and responses faster than 300 ms or slower than the time limit (accounting for 1.0% of the data) were discarded. One participant who produced a correct response in less than 75% of the trials was excluded. Participants’ mean RTs for correct responses and error rates were analyzed using an ANOVA with Congruency (Neutral vs. Incongruent) and Stimulus frequency (Low vs. High) as within-subject factors. The mean RTs and error rates are presented in Table 4, and raincloud plots of participants’ mean RTs are presented in Fig. 2.

**Table 4:** Mean response times (RTs) and percentage error rates (and corresponding 95% confidence intervals) in Experiment 2

Congruency	Stimulus frequency					
	RTs			Error rates		
	High	Low	Frequency effect	High	Low	Frequency effect
Neutral	726 [690, 763]	743 [705, 782]	17 [6, 28]	0.5 [0.3, 0.7]	0.7 [0.2, 1.1]	0.2 [−0.3, 0.7]
Incongruent	807 [761, 853]	831 [781, 882]	24 [9, 39]	1.9 [1.3, 2.5]	2.1 [1.3, 2.9]	0.2 [−0.8, 1.2]
Interference effect	81 [65, 97]	88 [67, 109]	7 [−9, 24]	1.4 [0.8, 2.0]	1.4 [0.4, 2.5]	0 [−1.1, 1.1]

*RTs*. There was a main effect of Congruency, *F*(1, 30) = 107.08, *MSE* = 2058.04, *p* <.001, ${\eta }_{p}^{2}$ =.781, indicating an interference effect, with faster responses to neutral than incongruent stimuli. There was also a main effect of Stimulus frequency, *F*(1, 30) = 17.49, *MSE* = 760.60, *p* <.001, ${\eta }_{p}^{2}$ =.368, indicating faster responses to high- than low-frequency stimuli. There was no interaction between Congruency and Stimulus frequency, however, *F*(1, 30) = 0.82, *MSE* = 505.86, *p* =.372, ${\eta }_{p}^{2}$ =.027, suggesting little if any difference between the frequency effects produced by neutral (17 ms, 95% CI [6, 28]) and incongruent stimuli (24 ms, 95% CI [9, 28]).

*Error rates*. There was a main effect of Congruency, *F*(1, 30) = 22.87, *MSE* <.001, *p* <.001, ${\eta }_{p}^{2}$ =.433, indicating an interference effect, with more accurate responses to neutral than incongruent stimuli. Neither of the other effects was significant, both *p*s ≥.426.

## Discussion

Experiment 2 produced direct evidence for the role of stimulus frequency in Stroop tasks. In both neutral and incongruent conditions, responses were faster for high- than low-frequency stimuli, an effect that can be explained by neither adaptive control nor contingency learning (but see footnote 1). In contrast, the effect seems to reflect a more general benefit for stimuli repeatedly encountered (Logan, 1988). More importantly, the demonstration of the effect’s existence in Experiment 2 supports the idea that a similar effect might have played a role in Experiment 1 as well. Specifically, because neutral stimuli were individually more frequent in the MN condition than in the MI condition, a stimulus-frequency effect would be able to explain why the former elicited faster responses than the latter, a pattern that would be otherwise difficult to explain. (A similar reasoning applies to incongruent stimuli, which were individually more frequent, and faster, in the MI condition than in the MN condition.)

On the other hand, although the stimulus-frequency manipulation was the same in the two experiments, the relevant contrasts were larger in Experiment 1 (22 ms, 95% CI [7, 38], for neutral stimuli, 49 ms, 95% CI [28, 70], for incongruent ones) than in Experiment 2 (17, 95% CI [6, 28], and 24 ms, 95% CI [9, 39], respectively). This fact would suggest that although stimulus frequency might explain the pattern with the neutral stimuli that emerged in Experiment 1, it would be unlikely to be able to explain the entire pattern presented by incongruent stimuli in that experiment.

## Experiment 3

In Experiment 3 the aim was to improve Experiment 1’s design by dissociating item-specific conflict frequency from stimulus frequency. To do so, we implemented an item-specific PN manipulation as in Experiment 1, but distinguishing two sets of distractors: an inducer set that, albeit necessary for the manipulation, involved a stimulus-frequency confound (as did all items in Experiment 1), and a diagnostic set that involved no stimulus-frequency confound because it involved neutral and incongruent stimuli presented with the same proportions and frequencies across MN and MI conditions. In such a design, following a similar logic to that applied to list-wide manipulations, diagnostic items provide the crucial piece of evidence for adjudicating a potential role for item-specific conflict adaptation. For the reasons discussed, this evidence should emerge in the form of an item-specific PN effect primarily driven by incongruent stimuli.

### Method

#### Participants

Reasoning that an item-specific PN effect controlled for stimulus frequency would likely be smaller than the one obtained in Experiment 1, we decided to double the sample size used in that experiment, i.e., from 32 to 64 participants. In order to determine how small an effect this sample allowed us to detect, we conducted a sensitivity analysis with G*Power using similar settings as those used for the previous experiments. The analysis revealed that this sample allowed detection of an effect as small as ${\eta }_{p}^{2}$ = 0.114 with a power of .80. Sixty-five (64 after exclusions, see below) students at the University of Western Ontario (age 19–33 years) participated for course credit. All participants reported being native English speakers and having normal or corrected-to-normal vision. None had participated in either of the previous experiments.

#### Materials

We used the same four color names and four letter strings used in the previous experiments as distractors and the same four colors as targets. Also as in the previous experiments, the four targets were divided in two sets (one including red and blue, which were only combined with GREEN, YELLOW, JJJJJ, and HHHHHH, and the other including green and yellow, which were only combined with RED, BLUE, FFF, and KKKK), and, as in Experiment 2, the distractors were also divided in two sets (one including RED, GREEN, FFF, and JJJJJ, and the other including BLUE, YELLOW, KKKK, and HHHHHH).

**Fig. 3:**
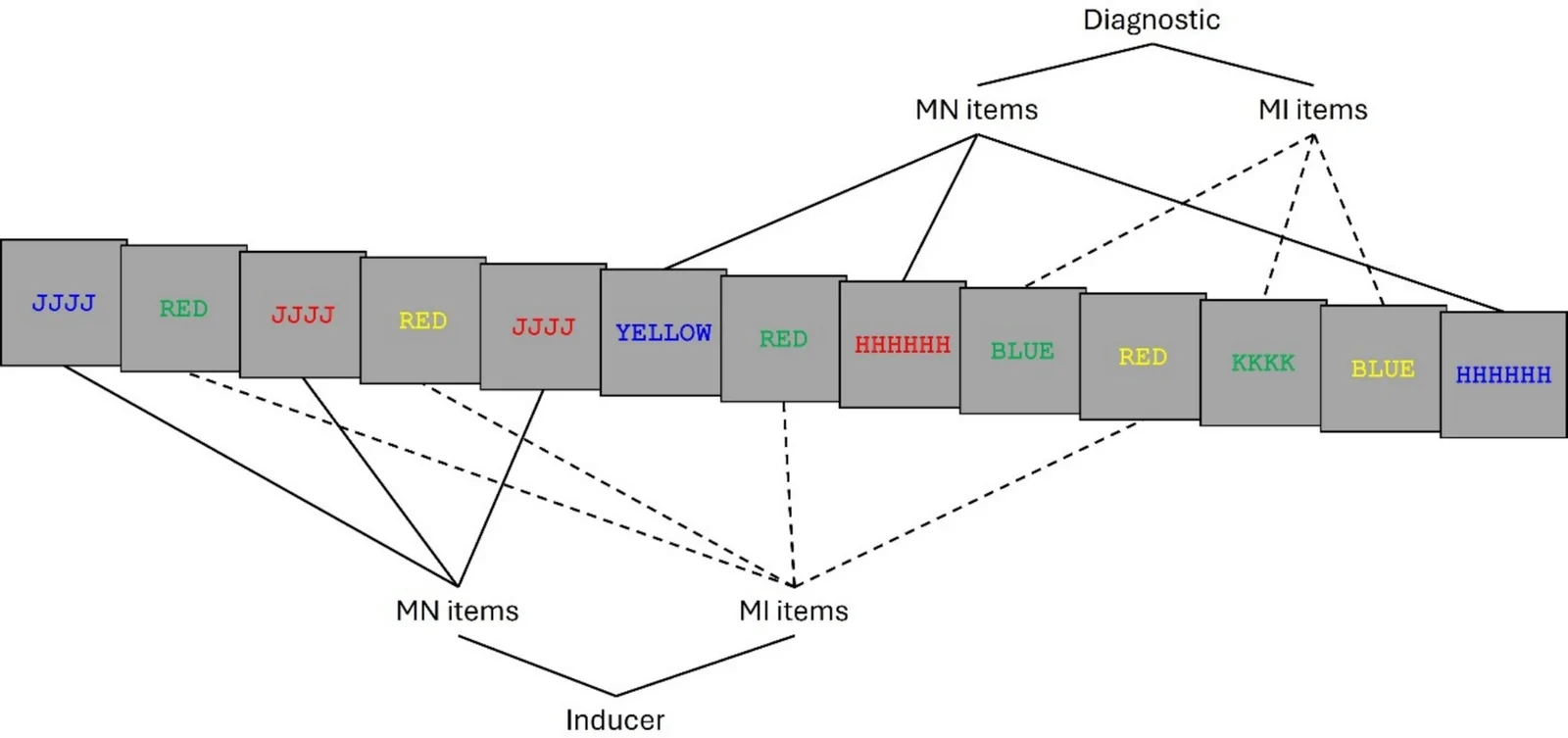
A representation of a possible trial sequence in Experiment 3. See the online version of this article for colors

Four versions of the experiment, counterbalanced across participants, were created. One of the versions is represented in Table 5 and in Fig. 3 in the form of a potential trial sequence. In each version, one of the two target sets (e.g., the one including the colors red and blue) served as the MN set and the other (e.g., the one including the colors green and yellow) served as the MI set. In addition, one of the two distractor sets (e.g., the one including RED, GREEN, FFF, and JJJJJ) served as the inducer set and the other (e.g., the one including BLUE, YELLOW, KKKK, and HHHHHH) served as the diagnostic set. In the MN set, each color was presented 54 times with the letter string and six times with the incongruent color name associated with that color in the inducer set (e.g., for the color red, JJJJJ and GREEN), and 18 times with the letter string and 18 times with the incongruent color name associated with that color in the diagnostic set (e.g., for the color red, HHHHHH and YELLOW). Conversely in the MI set, each color was presented six times with the letter string and 54 times with the incongruent color name associated with that color in the inducer set (e.g., for the color green, FFF and RED), and 18 times with the letter string and 18 times with the incongruent color name associated with that color in the diagnostic set (e.g., for the color green, KKKK and BLUE).

**Table 5:** Template for the frequency of target-distractor combinations in Experiment 3

		Distractor							
		Inducer				Diagnostic			
Set	Target	RED	GREEN	FFF	JJJJJ	BLUE	YELLOW	KKKK	HHHHHH
MN	Red		6		54		18		18
	Blue		6		54		18		18
MI	Green	54		6		18		18	
	Yellow	54		6		18		18	

*MN* Mostly Neutral, *MI* Mostly Incongruent

Overall, as in Experiment 1, the target-specific proportion of incongruent stimuli was 25% for MN colors and 75% for MI colors. Note, however, that this manipulation was confounded with stimulus frequency in the inducer set but not in the diagnostic set, because neutral and incongruent stimuli were presented with the same frequency for MN and MI colors in the diagnostic set. Finally, as in the previous experiments, the list-wide proportion of incongruent stimuli was 50%, with 192 incongruent and 192 neutral stimuli overall (384 stimuli in total), and the distractor-specific proportion of incongruent stimuli was 100% for color names and 0% for letter strings.

#### Procedure

The procedure was the same as in the previous experiments except for the stimulus set and the fact that the experiment was administered to 64 valid participants. Note that because the order of the stimuli was randomized, diagnostic and inducer items, either of the MN or MI kind, were intermixed, as represented in Fig. 3.

### Results

**Fig. 4:**
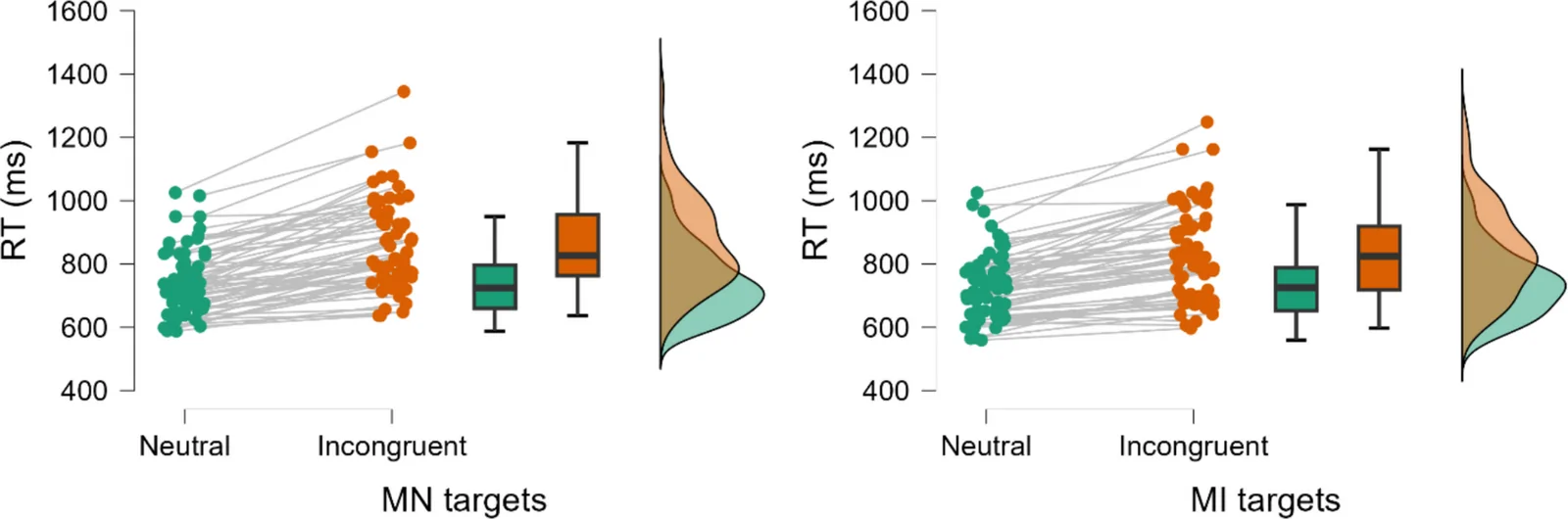
Raincloud plots of participants’ mean response times (RTs) for diagnostic items in Experiment 3. MN = Mostly Neutral; MI = Mostly Incongruent. See the online version of this article for colors

The data were pre-processed in the same way as in the previous experiments. Prior to the analyses, invalid trials due to technical failures and responses faster than 300 ms or slower than the time limit (accounting for 2.0% of the data) were discarded. One participant who produced a correct response in less than 75% of the trials was excluded. Separate analyses were conducted for inducer and diagnostic items, as is standard for designs that make this distinction (Braem et al., 2019). For both item types, participants’ mean RTs for correct responses and error rates were analyzed using an ANOVA with Congruency (Neutral vs. Incongruent) and Target type (Mostly neutral vs. Mostly incongruent) as within-subject factors. The mean RTs and error rates are presented in Table 6 for inducer items and in Table 7 for diagnostic items, and raincloud plots of participants’ mean RTs for diagnostic items (the crucial stimuli in the experiment) are presented in Fig. 4.

**Table 6:** Mean response times (RTs) and percentage error rates (and corresponding 95% confidence intervals) for inducer items in Experiment 3

	Target type					
	RTs			Error rates		
Congruency	MN	MI	Target type effect	MN	MI	Target type effect
Neutral	721 [697, 746]	751 [724, 778]	30 [16, 43]	0.9 [0.4, 1.3]	0.9 [0.2, 1.7]	0 [−0.8, 0.9]
Incongruent	870 [830, 910]	804 [771, 836]	−66 [−88, −46]	4.7 [2.6, 6.9]	2.7 [1.6, 3.7]	−2.0 [−4.2, 0.0]
Interference effect	149 [124, 175]	53 [37, 68]	−96 [−119, −74]	3.8 [1.7, 6.0]	1.8 [0.7, 2.8]	−2.0 [−4.3, 0.1]

*MN* Mostly Neutral, *MI* Mostly Incongruent

**Table 7:** Mean response times (RTs) and percentage error rates (and corresponding 95% confidence intervals) for diagnostic items in Experiment 3

	Target type					
	RTs			Error rates		
Congruency	MN	MI	Target type effect	MN	MI	Target type effect
Neutral	739 [713, 765]	733 [707, 759]	−6 [−17, 5]	1.4 [0.6, 2.2]	1.0 [0.5, 1.4]	−0.4 [−1.1, 0.2]
Incongruent	864 [828, 899]	837 [801, 872]	−27 [−42, −12]	4.0 [2.7, 5.3]	2.7 [1.8, 3.7]	−1.3 [−2.4, 0.0]
Interference effect	125 [104, 146]	104 [87, 121]	−21 [−34, −7]	2.6 [1.4, 3.7]	1.7 [0.9, 2.6]	−0.9 [−2.2, 0.6]

*MN* Mostly Neutral, *MI* Mostly Incongruent

#### Inducer items

*RTs*. There was a main effect of Congruency, *F*(1, 63) = 126.33, *MSE* = 5157.41, *p* <.001, ${\eta }_{p}^{2}$ =.667, indicating an interference effect, with faster responses to neutral than incongruent stimuli. There was also a main effect of Target Type, *F*(1, 63) = 7.36, *MSE* = 3011.24, *p* =.009, ${\eta }_{p}^{2}$ =.105, indicating slower responses to MN than MI targets overall. Congruency and Target type also interacted, *F*(1, 63) = 72.51, *MSE* = 2063.51, *p* <.001, ${\eta }_{p}^{2}$ =.535, reflecting an item-specific PN effect, with a larger interference effect for MN targets (149 ms, 95% CI [124, 175]) than for MI targets (53 ms, 95% CI [37, 68]). A simple main effect analysis revealed that, as in Experiment 1, this interaction resulted from both a speed-up for neutral stimuli appearing with MN versus MI targets, *t*(63) = −4.42, *p* <.001, ${\eta }_{p}^{2}$ =.237, and a slow-down for incongruent stimuli appearing with MN versus MI targets, *t*(63) = 6.29, *p* <.001, ${\eta }_{p}^{2}$ =.386.

*Error rates*. There was a main effect of Congruency, *F*(1, 63) = 19.87, *MSE* =.003, *p* <.001, ${\eta }_{p}^{2}$ =.240, indicating an interference effect, with more accurate responses to neutral than incongruent stimuli. Both the main effect of Target type, *F*(1, 63) = 3.00, *MSE* =.002, *p* =.088, ${\eta }_{p}^{2}$ =.045, and the interaction between Congruency and Target type, *F*(1, 63) = 3.74, *MSE* =.002, *p* =.058, ${\eta }_{p}^{2}$ =.056, were marginal, reflecting the fact that responses were less accurate to MN than MI targets, with this difference emerging virtually exclusively for incongruent stimuli, but neither of those effects were statistically significant.

#### Diagnostic items

*RTs*. There was a main effect of Congruency, *F*(1, 63) = 162.22, *MSE* = 5162.79, *p* <.001, ${\eta }_{p}^{2}$ =.720, indicating, once again, an interference effect, with faster responses to neutral than incongruent stimuli. There was also a main effect of Target Type, *F*(1, 63) = 8.12, *MSE* = 2132.63, *p* =.006, ${\eta }_{p}^{2}$ =.114, indicating slower responses to MN than MI targets overall. Most importantly, there was also an interaction between Congruency and Target type, *F*(1, 63) = 9.15, *MSE* = 741.84, *p* =.004, ${\eta }_{p}^{2}$ =.127, reflecting an item-specific PN effect, with a larger interference effect for MN targets (125 ms, 95% CI [104, 146]) than for MI targets (104 ms, 95% CI [87, 121]). A simple main effect analysis revealed that, as in Experiment 1 and in the inducer items in the present experiment, this interaction reflected a slow-down for incongruent stimuli appearing with MN versus MI targets, *t*(63) = 3.52, *p* <.001, ${\eta }_{p}^{2}$ =.164. Unlike in those previous situations, however, there was no speed-up for neutral stimuli appearing with MN versus MI targets in the present situation – indeed, there was a small (−6 ms, 95% CI [−17, 5]) and non-significant difference in the opposite direction, *t*(63) = 1.09, *p* =.280, ${\eta }_{p}^{2}$ =.018.

*Error rates*. There was a main effect of Congruency, *F*(1, 63) = 34.69, *MSE* =.001, *p* <.001, ${\eta }_{p}^{2}$ =.355, indicating an interference effect, with more accurate responses to neutral than incongruent stimuli. There was also a main effect of Target type, *F*(1, 63) = 5.94, *MSE* =.001, *p* =.018, ${\eta }_{p}^{2}$ =.086, indicating less accurate responses to MN than MI targets overall. Congruency and Target Type, however, did not interact, *F*(1, 63) = 1.22, *MSE* =.001, *p* =.274, ${\eta }_{p}^{2}$ =.019.

## Discussion

By distinguishing between inducer items involving a stimulus-frequency confound and diagnostic items not involving such a confound, Experiment 3 provided a clearer picture of the processes involved in our item-specific PN manipulation. Inducer items produced, in the latencies, a similar pattern to that in Experiment 1 (an experiment in which all of the stimuli involved the stimulus-frequency confound): an item-specific PN effect driven not only by a slow-down for incongruent stimuli with MN than MI targets but also a speed-up for neutral stimuli with MN than MI targets. As noted when discussing Experiment 1, the pattern for incongruent stimuli is compatible with both an item-specific conflict-adaptation process and a stimulus-frequency one, but the pattern for neutral stimuli is only compatible with the latter process. In contrast, diagnostic items also produced a (smaller) item-specific PN effect in the latencies, one driven exclusively by a slow-down for incongruent stimuli with MN than MI targets. This pattern is the type of pattern that is expected assuming item-specific conflict adaptation as the main process underlying performance for diagnostic items, items that allowed no contingency learning and were controlled on stimulus frequency.^3^ Hence, this result represents one of the clearest pieces of evidence thus far for a “pure” item-specific conflict-adaptation process, the type of process that, since Jacoby et al. (2003), item-specific PC manipulations have often been thought to demonstrate.

## General discussion

Jacoby’s work (Bugg et al., 2008, 2011a; Jacoby et al., 1999, 2003; Trainham et al., 1997; see also Lindsay & Jacoby, 1994) has had a profound impact on the Stroop literature, suggesting that the underlying process of many PC manipulations might be an item-specific one (Bugg et al., 2008) and that this process might be control-based in addition to contingency-based (Jacoby et al., 2003). An outstanding issue, however, is the role that conflict plays in driving item-specific adaptive control, i.e., is there such a thing as item-specific conflict adaptation?

Building on previous work of ours (Spinelli & Lupker, 2021), we attempted to answer that question by designing an item-specific Proportion-Neutral (PN) manipulation in a color-word Stroop task that, like Jacoby et al.’s (2003) item-specific Proportion-*Congruent* (PC) manipulation, involved a contrast between two sets of items differing in congruency proportion. Different from the original manipulation, however, no congruent items were included in the present manipulations in order to prevent the facilitation associated with those items from having an impact. Instead, we contrasted Mostly Neutral (MN) and Mostly Incongruent (MI) items defined by their target (i.e., color) identities in a “pure” conflict-frequency manipulation.

In Experiment 1, this contrast produced an item-specific PN effect similar to the item-specific PC effect typically observed in the standard manipulation: MN targets produced a larger interference effect than MI targets. This pattern, however, was due to both incongruent stimuli slowing down in the MN condition and neutral stimuli speeding up in the MN condition. While the former result is compatible with a conflict-adaptation process, the latter is not, because a conflict-adaptation process should have little, if any, consequence for stimuli involving neither conflict nor facilitation.

This result suggested that the observed pattern might be at least partially driven by the fact that our conflict-frequency manipulation was also a stimulus-frequency one. In Experiment 2, we assessed the potential impact of this confound by manipulating stimulus frequency independently from item-specific conflict frequency. The results confirmed that stimulus frequency does have a significant impact, with high-frequency stimuli eliciting faster responses than low-frequency stimuli in both incongruent and neutral conditions. This finding supported the idea that stimulus frequency likely contributed to the effects observed in Experiment 1.

Experiment 3, however, provided conclusive evidence that stimulus frequency was not the only process responsible for producing a PN effect in our manipulation, as an item-specific PN effect emerged both for items for which stimulus frequency was confounded with the congruency proportion manipulation (i.e., the inducer items) and for items for which there was no such confound (i.e., the diagnostic items). Indeed, for the latter items, the item-specific PN effect was entirely driven by incongruent stimuli being slower in the MN condition than in the MI condition, the type of pattern that would be expected based on a conflict-adaptation process.

Overall, these results are consistent with the notion that item-specific conflict adaptation is a tool at our disposal. Specifically, they suggest that individuals can use the conflict frequency associated with a specific target to reduce selectivity when the likelihood of conflict arising for that target is low (an MN target) and/or increase selectivity when the likelihood of conflict arising for that target is high (an MI target). As noted, this process is not one that can be prepared in advance of stimulus presentation (a proactive process) but must be one implemented upon stimulus presentation (a reactive process; Gonthier et al., 2016).

Of course, having established that individuals *can* use a tool does not imply that they *will* use it in all situations in which its use may be beneficial. Indeed, our situation might be special because, especially for diagnostic items in Experiment 3, participants could use no process other than item-specific conflict adaptation (i.e., item-nonspecific conflict adaptation, contingency learning, or stimulus frequency were not possible to use) to improve their performance.

A potential implication is that the use of such a process might be more limited in situations that do allow other processes to improve performance (Bugg, 2015; Bugg & Hutchison, 2013). Note, however, that our examination of this and similar accounts proposing a trade-off between adaptive control and more general processes (e.g., Bugg, 2014) suggests that a strong version of this type of account – the idea that adaptive-control processes would be *absent* when alternative processes are possible – is likely incorrect. The reason is that we have found evidence for both list-wide and item-specific adaptive control in situations that allowed contingency learning as an alternative process for minimizing interference in MI conditions – a type of situation in which, according to trade-off accounts, adaptive control would not be engaged (Spinelli & Lupker, 2020, 2023, 2024; Spinelli et al., 2022). Of course, these results are not inconsistent with a weaker version of trade-off accounts according to which reliance on adaptive control would be reduced when alternative processes are possible compared to when adaptive control is the last/only resort.

The more important point for present purposes, however, is that multiple processes – not limited to those of the adaptive-control kind – are likely involved in manipulations supposed to measure adaptive control (e.g., Schmidt, 2013a, 2019), and evidence exists that those processes can co-exist. Applied to the present situation, this notion of multiple processes co-existing in adaptive-control manipulations has at least two implications. The first is that, in principle, it seems possible that the item-specific conflict-adaptation process revealed by the present experiments would not be limited to the situations created by those experiments. Instead, it likely extends to other relevant situations, including the original item-specific PC manipulation (Jacoby et al., 2003).

The second implication is that, when manipulating congruency proportion in an item-specific fashion, researchers should be mindful that, unless specific actions are taken, variables other than congruency proportion are likely being manipulated at the same time. Many of these variables could have a confounding influence. Specifically, the stimulus-frequency effect revealed by our Experiment 2 suggests that stimulus frequency should be controlled when manipulating item-specific conflict frequency. Indeed, that finding likely has implications for any contrast between stimuli with unmatched frequency, as often occurs when contrasting infrequent neutral stimuli with more frequent congruent and incongruent ones (Spinelli & Lupker, 2025).

In sum, the present experiments provide compelling evidence for the existence of item-specific conflict adaptation, a process that, since Jacoby et al.’s (2003) seminal work, has been frequently assumed but not clearly demonstrated. However, as Jacoby et al. cautioned, item-specific adaptive control is likely not the only process item-specific congruency manipulations involve. Jacoby’s legacy lies precisely in this nuanced view of performance in adaptive-control paradigms, a view that researchers would be well advised to keep in mind.

## Supplementary Information

Below is the link to the electronic supplementary material.

Supplementary file1 (DOCX 55 KB)

## Data Availability

The datasets analyzed in the study are available on the Open Science Framework at https://osf.io/dfgkh/.

## Footnotes

1 While we presented contingency learning and stimulus frequency as separate processes here, it must be noted that Schmidt et al. (2020) proposed that the two might reflect essentially the same process, a broader contingency-learning process involving the acquisition of the association between a distractor and the (correct) response in the stimuli involving that distractor that are most frequently presented. By that definition of contingency learning, the present Experiment 1 could not be said to control for that process (Spinelli & Lupker,^ 2023^). The same is not true for the present Experiment 3, however.

2 Note that low-frequency distractors (each of which appeared 24 times) were also three times less frequent than high-frequency ones (each of which appeared 72 times). The implication is that our manipulation was not only a stimulus-frequency one but also a distractor-frequency one. Note, however, that in Experiment 1 low/high-frequency stimuli were also presented with low/high-frequency distractors. Therefore, the distinction between stimulus and distractor frequency was irrelevant for present purposes and will not be discussed further.

3 As a manipulation check, we examined immediate and episodic stimulus repetitions for diagnostic items in Experiment 3 and found no association of either type of repetition with the item-specific PN manipulation (for details, see the Online Supplementary Materials). This lack of an association implies that the item-specific PN effect on diagnostic items in Experiment 3 cannot be explained in terms of either immediate (Cochrane & Pratt, 2022) or episodic stimulus repetitions (Gallego et al., 2025).

## Code availability

The scripts used to perform the analyses in the study are available on the Open Science Framework https://osf.io/dfgkh/.

## Declarations

### Open practices statement

The data and JASP scripts used for the analyses are publicly available at https://osf.io/dfgkh/. The experiment was not preregistered.
