# Supplementary material for: Focusing on conflict in item-specific adaptive control: Insights from a proportion-neutral manipulation
Source: Mem Cognit. 2026 Apr 8;54(7):2615–32. doi: 10.3758/s13421-026-01878-2 (PMC13751689; doi:10.3758/s13421-026-01878-2)
Supplement: Supplementary file 1 [file 13421_2026_1878_MOESM1_ESM.docx]

Supplementary materials for:

Focusing on conflict in item-specific adaptive control: Insights from a proportion-neutral manipulation

**Experiment 1**

Reported here are the analyses we conducted in an attempt to evaluate the idea that a stimulus-frequency confound might have contributed to the item-specific Proportion-Neutral (PN) effect obtained in Experiment 1. To that end, we used two proxies for stimulus-frequency effects.

First, we reasoned that the primary impact of a stimulus-frequency confound would be to increase the likelihood of the impact of immediate stimulus repetitions for conditions associated with a high frequency of such repetitions (the high-frequency stimuli, i.e., the neutral stimuli in the Mostly Neutral (MN) condition and the incongruent stimuli in the Mostly Incongruent (MI) condition). The result would be an increased item-specific PN effect (Cochrane & Pratt, 2022). In line with research in feature binding (e.g., Schmidt et al., 2020), we distinguished between target and distractor repetitions, so that a trial could involve a target repetition and a distractor repetition (TRDR, e.g., RED in green following RED in green), a target repetition and a distractor alternation (TRDA, e.g., RED in green following KKKK in green), a target alternation and a distractor repetition (TADR, e.g., RED in green following RED in yellow), or a target alternation and a distractor alternation (TADA, e.g., RED in green following KKKK in yellow).

Second, building on the idea that a distractor can be used to retrieve episodes of past trials containing that distractor (Giesen et al., 2020), we focused on whether the most recent episode containing the current-trial distractor (e.g., the word RED presented in green) also contained the current-trial target (e.g., RED in green, i.e., a target repetition and a distractor repetition—TRDR) or another target (e.g., RED in yellow, i.e., a target alternation and a distractor repetition—TADR). (To avoid confusion, in the following, we call these situations “episodic repetitions” even though what we refer to as “the most recent episode” could be as recent as the last trial, i.e., an immediate repetition. Typically, however, such episodes are more remote.) Again, our reasoning here was that episodic repetitions would be more frequent for conditions associated with a high stimulus frequency (i.e., neutral stimuli in the MN condition and incongruent stimuli in the MI condition) with the result being an increased item-specific PN effect (Gallego et al., 2025).

For both immediate and longer-term episodic repetitions, we proceeded as follows. First, we determined whether there was an association between those repetitions and the four conditions involved in our experiment (MN neutral, MN incongruent, MI neutral, and MI incongruent). Second, we determined whether those repetitions impacted performance independently from the experimental manipulation. Third, we re-analyzed the data for the experimental manipulation using subsets of the data for which the impact of the relevant repetitions was reduced or null. Finally, we conducted an exploratory analysis with linear mixed-effects models in order to examine the impact of the item-specific PN manipulation along with that of repetitions.

*Association between conditions and repetition type*

As noted, we first determined whether there was an association between immediate/episodic repetitions and the four conditions involved in our experiment (MN neutral, MN incongruent, MI neutral, and MI incongruent). In order to do so, we counted the number of repetitions of each type across conditions and participants for all trials excluding the first one in a block (for immediate repetitions) and excluding the first presentation of each distractor in a block (for episodic repetitions) as first presentations cannot be repetitions. We then used those counts to conduct a Chi-square test of independence between Condition (coded as MN neutral vs. MN incongruent vs. MI neutral vs. MI incongruent) and Repetition type (coded as TRDR vs. TRDA vs. TADR vs. TADA for immediate repetitions and as TRDR vs. TADR for episodic repetitions). The relevant contingency tables are reported in Table S1 for immediate repetitions and Table S2 for episodic repetitions. Adjusted Pearson residuals are reported in each cell in order to help interpret the results of the Chi-square test of independence, which was significant in both cases (*χ*(9) = 364.58, *p* < .001, for immediate repetitions, and *χ*(3) = 294.83, *p* < .001, for episodic repetitions).

In both tables, the totals for Condition reflect the nature of the manipulation (i.e., more observations for MN neutral and MI incongruent than for MN incongruent and MI neutral). Focusing on immediate repetitions (Table S1), the totals for Repetition Type are also a reflection of the nature of the manipulation, because, with 16 possible stimuli (see Table 1 in the main text), it would be very likely to have a full repetition (TRDR, the most frequent case) and quite unlikely to have a full alternation (TADA, the least frequent case). In addition, because there were 4 targets and 8 distractors, it was more likely for the target to repeat without the distractor also repeating (TRDA) than it was for the distractor to repeat without the target also repeating (TADR).

More importantly, Condition and Repetition type were associated for immediate repetitions, with adjusted Pearson residuals revealing that all cells contributed to this association (all *r*s > 1.96). However, the contribution seemed to be more pronounced for full repetitions (TRDR), which were much more frequent than expected in the MN neutral and MI incongruent conditions (i.e., the high-frequency conditions) and much less frequent than expected in the MN incongruent and MI neutral conditions (i.e., the low-frequency conditions). Indeed, there were no full repetitions at all in the MN incongruent condition and only 3 in the MI neutral condition.

Table S1

*Contingency table (with adjusted Pearson residuals) of Condition and Repetition type for immediate repetitions in Experiment 1*

|  | Repetition type | | | |  |
| --- | --- | --- | --- | --- | --- |
| Condition | TRDR | TRDA | TADR | TADA | Total |
| MN neutral | 266 (5.18) | 758 (−3.06) | 426 (4.96) | 3138 (−2.68) | 4588 |
| MN incongruent | 0 (−9.12) | 362 (6.31) | 51 (−6.89) | 1116 (2.88) | 1529 |
| MI neutral | 3 (−8.72) | 346 (5.18) | 44 (−7.60) | 1135 (4.05) | 1528 |
| MI incongruent | 286 (7.01) | 721 (−4.79) | 425 (4.94) | 3147 (−2.06) | 4579 |
| Total | 555 | 2187 | 946 | 8536 | 12224 |

Note. MN = Mostly Neutral; MI = Mostly Incongruent; TRDR = Target Repetition and Distractor Repetition; TRDA = Target Repetition and Distractor Alternation; TADR = Target Alternation and Distractor Repetition; TADA = Target Alternation and Distractor Alternation.

Inspection of episodic repetitions (Table S2) revealed that, first, in the most recent episode in which the distractor matched that of the current trial, target alternations were overall more common than target repetitions. Second and more importantly, in this case as well, Condition and Repetition type were associated, with, again, all cells contributing to this association (all *r*s > 1.96). In particular, target repetitions were much more frequent than expected for the MN neutral and MI incongruent conditions (i.e., the high-frequency conditions) and much less frequent than expected in the MN incongruent and MI neutral conditions (i.e., the low-frequency conditions), with the reverse pattern for target alternations.

Table S2

*Contingency table (with adjusted Pearson residuals) of Condition and Repetition type for episodic repetitions in Experiment 1*

|  | Repetition type | |  |
| --- | --- | --- | --- |
| Condition | TRDR | TADR | Total |
| MN neutral | 1852 (7.00) | 2628 (−7.00) | 4480 |
| MN incongruent | 343 (−10.74) | 1065 (10.74) | 1408 |
| MI neutral | 325 (−11.80) | 1083 (11.80) | 1408 |
| MI incongruent | 1879 (8.06) | 2601 (−8.06) | 4480 |
| Total | 4399 | 7377 | 12224 |

Note. MN = Mostly Neutral; MI = Mostly Incongruent; TRDR = Target Repetition and Distractor Repetition; TADR = Target Alternation and Distractor Repetition.

*Impact of repetitions*

Having established an association between the conditions in our manipulation and the type of immediate/episodic repetitions that could occur, we determined whether those repetitions had an impact on participants’ performance. To do so, we first excluded, for immediate repetitions, the initial trial in a block and any trial following an error or invalid response. For episodic repetitions, we also excluded the first presentation of each distractor in a block. Next, participants’ mean RTs for correct responses and error rates were analyzed for immediate repetitions, using an ANOVA with Target repetition type (Target repetition vs. Target alternation) and Distractor repetition type (Distractor repetition vs. Distractor alternation) as within-subject factors. For episodic repetitions, we used a paired-samples t-test comparing target repetitions and target alternations. Congruency (Neutral vs. Incongruent) and Target type (MN vs. MI) were not included in either analysis because, first, as revealed by the previous analysis, some cells would have had very few (or no) observations, particularly for immediate repetitions. Second, the point of the analysis was to establish whether immediate/episodic repetitions would matter regardless of the condition they appeared in. Thus, we sought to determine whether the data from Experiment 1 would allow a replication of the standard results in this type of analysis, that is, for immediate repetitions, responses are faster for target repetitions than for response alternations, but especially so when the distractor also repeats (i.e., with full repetitions; Frings et al., 2007), and, for episodic repetitions, responses are faster when, in the most recent episode in which the distractor matched that of the current trial, the target also matched (vs. mismatched) that of the current trial (Giesen et al., 2020).

The results were consistent with these ideas. For immediate repetitions, the mean RTs and error rates are presented in Table S3. In the RTs, there was a main effect of Target repetition type, *F*(1, 31) = 83.03, *MSE* = 2534.54, *p* < .001, $\eta_{p}^{2}$ = .728, indicating faster responses with target repetitions than with target alternations. There was also a main effect of Distractor repetition type, *F*(1, 31) = 47.27, *MSE* = 748.48, *p* < .001, $\eta_{p}^{2}$ = .604, indicating faster responses with distractor repetitions than with distractor alternations. There was also a significant interaction between the two, *F*(1, 31) = 8.17, *MSE* = 686.05, *p* = .008, $\eta_{p}^{2}$ = .209, reflecting the fact that the target-repetition effect was larger for distractor repetitions (94 ms) than for distractor alternations (68 ms). Note that distractor repetitions sped up responses both with target repetitions, *t*(31) = 6.95, *p* < .001, $\eta_{p}^{2}$ = .609, and with target alternations, *t*(31) = 2.99, *p* = .005, $\eta_{p}^{2}$ = .224, whereas it is more typical for distractor repetitions to produce, if anything, a small slow-down with target alternations (Schmidt et al., 2020). In the error rates, the only significant effect was that of Target repetition type, *F*(1, 31) = 5.76, *MSE* = .001, *p* = .023, $\eta_{p}^{2}$ = .157, indicating more accurate responses for target repetitions than for target alternations (other *p*s ≥ .116).

Table S3

*Mean RTs and percentage error rates (and corresponding 95% confidence intervals) for immediate target and distractor repetitions/alternations in Experiment 1*

|  | Target repetition type | | | | | |
| --- | --- | --- | --- | --- | --- | --- |
|  | RTs | |  | Error rates | |  |
| Distractor repetition type | Repetition | Alternation | Target repetition effect | Repetition | Alternation | Target repetition effect |
| Repetition | 679 [642, 715] | 773 [736, 810] | 94 [73, 116] | 0.5 [−0.1, 1.1] | 1.9 [0.6, 3.2] | 1.4 [0.1, 2.6] |
| Alternation | 725 [687, 763] | 793 [751, 835] | 68 [48, 87] | 1.2 [0.6, 1.7] | 1.8 [1.0, 2.5] | 0.6 [0.1, 1.1] |
| Distractor repetition effect | 46 [33, 60] | 20 [6, 34] | −26 [−45, −8] | 0.7 [0.0, 1.3] | −0.1 [−0.9, 0.7] | −0.8 [−1.7, 0.2] |

For episodic repetitions, the mean RTs and error rates are presented in Table S4. When the most recent episode containing the same distractor as that contained in the current trial also contained a target repetition (vs. alternation) responses were significantly faster, *t*(31) = 5.28, *p* < .001, $\eta_{p}^{2}$ = .474, but not significantly more accurate, *t*(31) = 1.18, *p* = .248, $\eta_{p}^{2}$ = .043. In sum, the evidence supported the idea that both immediate and episodic repetitions improved performance.

Table S4

*Mean RTs and percentage error rates (and corresponding 95% confidence intervals) for episodic repetitions in Experiment 1*

| Episodic target repetition type | RTs | Error rates |
| --- | --- | --- |
| Repetition | 760 [723, 798] | 1.4 [0.8, 2.0] |
| Alternation | 781 [740, 822] | 1.7 [0.9, 2.6] |
| Target repetition effect | 21 [13, 29] | 0.3 [−0.3, 0.9] |

*Removing the impact of repetitions*

The analyses thus far suggest that the conditions used in Experiment 1 were associated with the different types of immediate and episodic repetitions that can occur in experiments involving targets and distractors, and that these repetitions had an impact on performance similar to that typically found in the literature. Specifically, for immediate repetitions, both target and distractor repetitions had a facilitating effect, but especially so when they co-occurred. Similarly, for episodic repetitions, there was a facilitating effect when the most recent episode containing the current-trial distractor also contained the current-trial target. In this analysis, we attempted to control for this impact on the item-specific PN manipulation implemented in Experiment 1 by re-analyzing the data in the same way as in the main text (i.e., with Congruency and Target type as within-subject factors) by focusing on subsets of data in which the impact of repetitions would be reduced.

For immediate repetitions, we removed both target and distractor repetitions, i.e., we analyzed only trials involving target and distractor alternations (full alternations). The mean RTs and error rates are presented in Table S5. The results were very similar to the main-text analysis. Specifically, in the RTs, there was a significant main effect of Congruency, *F*(1, 31) = 173.99, *MSE* = 2015.33, *p* < .001, $\eta_{p}^{2}$ = .849, a marginal main effect of Target Type, *F*(1, 31) = 3.52, *MSE* = 1799.15, *p* = .070, $\eta_{p}^{2}$ = .102, and a significant interaction between Congruency and Target type, *F*(1, 31) = 28.25, *MSE* = 1535.63, *p* < .001, $\eta_{p}^{2}$ = .477. The latter reflected the same item-specific PN effect as the main-text analysis, with a simple main effect analysis revealing that, also as in the main-text analysis, this interaction resulted from both a speed-up for neutral stimuli appearing with MN vs. MI targets, *t*(31) = −2.40, *p* = .022, $\eta_{p}^{2}$ = .157, and a slow-down for incongruent stimuli appearing with MN vs. MI targets, *t*(31) = 4.67, *p* < .001, $\eta_{p}^{2}$ = .413. In the error rates, the only significant effect was the main effect of Congruency, *F*(1, 31) = 19.22, *MSE* = .001, *p* < .001, $\eta_{p}^{2}$ = .383 (other *p*s ≥ .585).

Table S5

*Mean RTs and percentage error rates (and corresponding 95% confidence intervals) in Experiment 1 for trials* involving target and distractor alternations *in immediate repetitions (full alternations)*

|  | Target type | | | | | |
| --- | --- | --- | --- | --- | --- | --- |
|  | RTs | |  | Error rates | |  |
| Congruency | MN | MI | Target type effect | MN | MI | Target type effect |
| Neutral | 739 [701, 777] | 762 [713, 810] | 23 [3, 42] | 0.7 [0.2, 1.3] | 0.7 [0.1, 1.3] | 0 [−0.8, 0.7] |
| Incongruent | 880 [829, 932] | 830 [787, 872] | −50 [−73, −29] | 3.3 [1.3, 5.2] | 2.8 [1.8, 3.7] | −0.5 [−2.1, 1.2] |
| Interference effect | 141 [117, 166] | 68 [50, 85] | −83 [−102, −45] | 2.6 [0.8, 4.2] | 2.1 [1.1, 3.1] | −0.5 [−2.2, 1.3] |

*Note*. MN = Mostly Neutral; MI = Mostly Incongruent.

For episodic repetitions, we removed the trials for which the most recent episode contained both the current-trial distractor and the current-trial target, i.e., we analyzed only trials involving a target alternation from the most recent episode. The mean RTs and error rates are presented in Table S6. Again, the results were very similar to the main-text analysis. Specifically, in the RTs, there was a significant main effect of Congruency, *F*(1, 31) = 158.81, *MSE* = 2134.25, *p* < .001, $\eta_{p}^{2}$ = .837, a marginal main effect of Target Type, *F*(1, 31) = 2.97, *MSE* = 2020.00, *p* = .087, $\eta_{p}^{2}$ = .102, and a significant interaction between Congruency and Target type, *F*(1, 31) = 26.34, *MSE* = 953.46, *p* < .001, $\eta_{p}^{2}$ = .459. The latter reflected a similar item-specific PN effect pattern as the main-text analysis, as the simple main effect analysis revealed that this interaction resulted from a slow-down for incongruent stimuli appearing with MN vs. MI targets, *t*(31) = 3.74, *p* < .001, $\eta_{p}^{2}$ = .310. There was also numerical tendency for a speed-up for neutral stimuli appearing with MN vs. MI targets, however, this tendency did not reach significance in this case, *t*(31) = −1.83, *p* = .077, $\eta_{p}^{2}$ = .098. In the error rates, the only significant effect was the main effect of Congruency, *F*(1, 31) = 15.16, *MSE* = .001, *p* < .001, $\eta_{p}^{2}$ = .328 (other *p*s ≥ .178).

Table S6

*Mean RTs and percentage error rates (and corresponding 95% confidence intervals) in Experiment 1 for trials involving target alternations in episodic repetitions*

|  | Target type | | | | | |
| --- | --- | --- | --- | --- | --- | --- |
|  | RTs | |  | Error rates | |  |
| Congruency | MN | MI | Target type effect | MN | MI | Target type effect |
| Neutral | 730 [692, 768] | 745 [702, 787] | 15 [−2, 30] | 0.7 [0.1, 1.3] | 0.7 [0.1, 1.2] | 0 [−0.8, 0.7] |
| Incongruent | 861 [810, 913] | 820 [777, 862] | −41 [−64, −19] | 3.6 [1.4, 5.9] | 2.5 [1.4, 3.6] | −1.1 [−2.9, 0.7] |
| Interference effect | 131 [109, 153] | 75 [57, 93] | −56 [−78, −34] | 2.9 [1.1, 4.8] | 1.8 [0.8, 2.9] | −1.1 [−2.7, 0.5] |

*Note*. MN = Mostly Neutral; MI = Mostly Incongruent.

In sum, controlling for the impact of immediate repetitions in Experiment 1 by restricting the analysis to full alternations did not eliminate the item-specific PN effect in the latencies. Note, however, that the effect size for that effect went from $\eta_{p}^{2}$ = .560 before that control was applied to $\eta_{p}^{2}$ = .477 after it was applied, and the effect size for the speed-up for neutral stimuli appearing with MN vs. MI targets went from $\eta_{p}^{2}$ = .214 to $\eta_{p}^{2}$ = .157. Although this reduction might reflect the reduced number of observations in the control analysis and/or the higher variability associated with full alternations, it is consistent with the idea that immediate repetitions might have provided a small contribution to the item-specific PN effect obtained in Experiment 1.

*Examining the impact of the item-specific PN manipulation along with that of repetitions*

The analyses thus far considered the impact of the item-specific PN manipulation separately from that of immediate/episodic repetitions. That is, in no analysis were the predictors relevant to the item-specific PN manipulation (i.e., Congruency and Target type) examined along with the predictors relevant to repetitions (e.g., for immediate repetitions, Target repetition and Distractor repetition). In fact, a simultaneous examination of these predictors was, in many cases, impossible in the context of mean-based ANOVAs because some cells had very few or no observations (see Table S1). However, this examination is possible in the context of mixed-effects models, which control for variability associated with individual participants and/or items (the random effects) and allow analysis at the trial level even when some combinations of the specified predictors (the fixed effects) are infrequent or absent (the interpretation in these cases, however, must still be cautious).

Based on these considerations, in this final analysis, we used linear mixed-effects models (LMMs) to conduct an exploratory examination of the impact of the item-specific PN manipulation along with that of immediate/episodic repetitions. We focused on RTs since the previous analyses indicated a stronger impact of both the item-specific PN manipulation and immediate/episodic repetitions on that measure. All analyses were conducted in R version 4.2.2 (R Core Team, 2022), with the *lmerTest* package, version 3.1-3 (Kuznetsova et al., 2017), used to fit the models and obtain probability estimates, the rsq package, version 2.5 (Zhang, 2022), used to get an estimate of $R^{2}$ (i.e., the proportion of total variance explained by the model), and the *emmeans* package, version 1.7.5 (Lenth, 2022), and the ggeffects package, version 1.0.1 (Lüdecke, 2018), used to conduct follow-up analyses. R-default treatment contrasts were changed to sum-to-zero contrasts (i.e., contr.sum) to help interpret lower-order effects in the presence of higher-order interactions.

We conducted three analyses. All three analyses were based on RTs from trials in which both the current response and the previous response were correct and that did not involve the first presentation of a distractor in a block. In all three analyses, the models included random intercepts for participants and the target stimuli (i.e., colors). Concerning fixed effects, the first analysis, serving as baseline (i.e., as a parallel to the analysis reported in the main text), included the fixed effects of Congruency (Neutral vs. Incongruent) and Target type (MN vs. MI), with their main effects and their interaction. The second analysis, focusing on immediate repetitions, not only included the baseline fixed effects but also included the fixed effects of Target repetition type (Target repetition vs. Target alternation from the previous trial) and Distractor repetition type (Distractor repetition vs. Distractor alternation from the previous trial), with their main effects and their interaction. Similarly, the third analysis, focusing on episodic repetitions, included, in addition to the baseline fixed effects, the fixed effect of Episodic target repetition type (Target repetition vs. Target alternation from the most recent “episode”, i.e., the most recent trial containing the same distractor contained in the current trial), the same effect examined in the previous analyses. In addition, this analysis also included the fixed effect of Episode distance, i.e., the distance of the most recent episode from the current trial, coded as a continuous predictor (with 1 meaning a one-trial distance, 2 a two-trial distance, etc.), as well as the interaction between this predictor and the Episodic target repetition type predictor. The reason for this additional predictor was that previous research indicated that, while a target repetition from the most recent episode facilitates performance, this facilitation decreases as the distance of that episode from the current trial increases (Gallego et al., 2025; see also Giesen et al., 2020). The distance predictor was z-transformed before the analyses as recommended for continuous predictors in mixed-effects models (Baayen, 2008).

Note that, in the latter two analyses, no interaction between the baseline predictors (i.e., Congruency and Target Type) and the repetition-related predictors was included because the interest was in whether the mere inclusion of the repetition-related predictors would influence the estimate for the item-specific PN effect, i.e., the interaction between Congruency and Target type. Specifically, based on the idea that stimulus frequency was confounded with the item-specific PN manipulation in Experiment 1, we expected a reduction for the estimate of that interaction in the analyses including the repetition-related predictors (for similar logic, see, e.g., Rothermund et al., 2022).

The results of all three analyses are reported in Table S7. The baseline model replicated the pattern of results reported in the main text—an item-specific PN effect. The immediate-repetitions model also replicated the results presented in the main text as well as those presented above, with both target and distractor repetitions having a facilitatory effect, with those effects being overadditive (i.e., full repetitions produced particularly fast responses). The episodic-repetitions model also replicated the results from the main text as well as showing, as reported above, that responses were overall faster when the target repeated from the most recent episode containing the same distractor as that contained in the current trial. In addition, consistent with previous findings (Gallego et al., 2025), this effect was modulated by the distance of the most recent episode, with the estimated effect being as large as 27 ms at distance 1 (i.e., when the most recent trial was the previous trial) and essentially zero at distance 20. Note also that both the immediate-repetitions and episodic-repetitions models outperformed the baseline model in goodness of fit, as measured by the Aikake Information Criterion (AIC), the Bayesian Information Criterion (BIC), and $R^{2}$.

Two main points emerge from these analyses. As noted just above, the crucial item-specific PN effect was significant in both models that included a repetition predictor. Second, even though the item-specific PN effect was significant in all models, the estimate for this effect was larger in the baseline model, β = −18.62, compared to when either the predictors relevant to immediate repetitions were added, β = −17.26, or the predictors relevant to episodic repetitions were added, β = −12.15. Therefore, consistent with the ANOVA results reported above, these results provide some support to the idea that stimulus frequency might have contributed to the item-specific PN effect in Experiment 1.

Table S7

*Linear Mixed-Effects Model results for the models with baseline and repetition-related predictors for Experiment 1*

|  | Goodness of fit | | | Model parameters | | | | |
| --- | --- | --- | --- | --- | --- | --- | --- | --- |
| Model | AIC | BIC | $R^{2}$ | Fixed effect | β | *SE* | *t* | *p* |
| Baseline | 147,366 | 147,417 | .3233 | Congruency | 50.09 | 1.93 | 25.90 | < .001 |
|  |  |  |  | Target type | −6.38 | 1.93 | −3.30 | < .001 |
|  |  |  |  | Congruency × Target type | −18.62 | 1.93 | −9.63 | < .001 |
| Immediate repetitions | 146,956 | 147,029 | .3475 | Congruency | 50.17 | 1.90 | 26.42 | < .001 |
|  |  |  |  | Target type | −6.62 | 1.90 | −3.48 | < .001 |
|  |  |  |  | Congruency × Target type | −17.26 | 1.93 | −8.96 | < .001 |
|  |  |  |  | Target repetition type | 40.44 | 2.58 | 15.71 | < .001 |
|  |  |  |  | Distractor repetition type | 12.63 | 2.61 | 4.84 | < .001 |
|  |  |  |  | Target repetition type × Distractor repetition type | −5.06 | 2.58 | −2.04 | .041 |
| Episodic repetitions | 147,317 | 147,390 | .3260 | Congruency | 50.05 | 1.93 | 25.93 | < .001 |
|  |  |  |  | Target type | −6.27 | 1.93 | −3.25 | .001 |
|  |  |  |  | Congruency × Target type | −12.15 | 2.44 | −4.99 | < .001 |
|  |  |  |  | Episodic target repetition type | 9.01 | 1.73 | 5.19 | < .001 |
|  |  |  |  | Episode distance | 8.10 | 2.06 | 3.92 | < .001 |
|  |  |  |  | Episodic target repetition type × Episode distance | −5.51 | 1.70 | −3.30 | < .001 |

**Experiment 3**

In Experiment 3, our item-specific PN manipulation was designed to control for any differential impact of stimulus frequency on the processing of the diagnostic stimuli across conditions. As a manipulation check, here we report the contingency tables showing that, for diagnostic stimuli, there is indeed no association between our four experimental conditions (MN neutral, MN incongruent, MI neutral, and MI incongruent) and either of the two proxies for stimulus frequency that we have considered for Experiment 1, i.e., immediate repetitions of the target and/or the distractor from the previous trial (Table S8) and episodic repetitions of the target from the most recent trial containing the same distractor as that contained in the current trial (Table S9). A Chi-square test of independence confirmed that there was no association between Condition and Repetition Type for either immediate repetitions, *χ*(9) = 9.93, *p* = .356, or episodic repetitions, *χ*(3) = 1.43, *p* = .700.

Table S8

*Contingency table of Condition and Repetition type for immediate repetitions for diagnostic stimuli in Experiment 3*

|  | Repetition type | | | |  |
| --- | --- | --- | --- | --- | --- |
| Condition | TRDR | TRDA | TADR | TADA | Total |
| MN neutral | 66 | 495 | 105 | 1663 | 2329 |
| MN incongruent | 76 | 428 | 117 | 1706 | 2327 |
| MI neutral | 67 | 481 | 112 | 1662 | 2322 |
| MI incongruent | 72 | 497 | 100 | 1660 | 2329 |
| Total | 281 | 1901 | 434 | 6691 | 9307 |

Note. MN = Mostly Neutral; MI = Mostly Incongruent; TRDR = Target Repetition and Distractor Repetition; TRDA = Target Repetition and Distractor Alternation; TADR = Target Alternation and Distractor Repetition; TADA = Target Alternation and Distractor Alternation.

Table S9

*Contingency table of Condition and Repetition type for episodic repetitions for diagnostic stimuli in Experiment 3*

|  | Repetition type | |  |
| --- | --- | --- | --- |
| Condition | TRDR | TADR | Total |
| MN neutral | 925 | 1285 | 2210 |
| MN incongruent | 914 | 1296 | 2210 |
| MI neutral | 930 | 1280 | 2210 |
| MI incongruent | 894 | 1316 | 2210 |
| Total | 3663 | 5177 | 8840 |

Note. MN = Mostly Neutral; MI = Mostly Incongruent; TRDR = Target Repetition and Distractor Repetition; TADR = Target Alternation and Distractor Repetition.

References

Baayen, R. H. (2008). Analyzing linguistic data: A practical introduction to statistics using R. Cambridge: Cambridge University Press. https://doi.org/10.1017/CBO9780511801686

Cochrane, B. A., & Pratt, J. (2022). The item-specific proportion congruency effect can be contaminated by short-term repetition priming. *Attention, Perception, & Psychophysics*, *84*(1), 1–9. https://doi.org/10.3758/s13414-021-02403-0

Frings, C., Rothermund, K., & Wentura, D. (2007). Distractor repetitions retrieve previous responses to targets. *Quarterly Journal of Experimental Psychology*, *60*, 1367–1377. https://doi.org/10.1080/17470210600955645

Gallego, D., Méndez, C., & Jiménez, L. (2025). Contingency learning and episodic contributions to the item-specific proportion congruent effect. *Quarterly Journal of Experimental Psychology*, *78*(5), 927–945. https://doi.org/10.1177/17470218231208664

Giesen, C. G., Schmidt, J. R., & Rothermund, K. (2020). The law of recency: An episodic stimulus-response retrieval account of habit acquisition. *Frontiers in Psychology*, *10*, 2927. https://doi.org/10.3389/fpsyg.2019.02927

Kuznetsova, A., Brockhoff, P. B., & Christensen, R. H. (2017). lmerTest package: tests in linear mixed effects models. *Journal of Statistical Software*, *82*, 1–26. https://doi.org/10.18637/jss.v082.i13

Lenth, R. (2022). emmeans: Estimated Marginal Means, aka Least-Squares Means. https://CRAN.R-project.org/package=emmeans

Lüdecke, D. (2018). ggeffects: Tidy Data Frames of Marginal Effects from Regression Models. Journal of Open Source Software, 3, 772. https://doi.org/10.21105/joss.00772

R Core Team (2022). *R: A language and environment for statistical computing*. R Foundation for Statistical Computing, Vienna, Austria. https://www.R-project.org/

Rothermund, K., Gollnick, N., & Giesen, C. G. (2022). Accounting for proportion congruency effects in the Stroop task in a confounded setup: Retrieval of stimulus-response episodes explains it all. *Journal of Cognition*, *5*(1), 39. https://doi.org/10.5334/joc.232

Schmidt, J. R., Giesen, C. G., & Rothermund, K. (2020). Contingency learning as binding? Testing an exemplar view of the colour-word contingency learning effect. *Quarterly Journal of Experimental Psychology*, *73*, 739–761. https://doi.org/10.1177/1747021820906397

Zhang, D. (2022). *rsq: R-Squared and Related Measures*. R package version 2.5. https://CRAN.R-project.org/package=rsq
